# BiVO_4_ Photoanodes Enhanced with Metal Phosphide Co‐Catalysts: Relevant Properties to Boost Photoanode Performance

**DOI:** 10.1002/smll.202306757

**Published:** 2023-10-06

**Authors:** Junyi Cui, Matyas Daboczi, Zhenyu Cui, Mengjun Gong, Joseph Flitcroft, Jonathan Skelton, Stephen C. Parker, Salvador Eslava

**Affiliations:** ^1^ Department of Chemical Engineering and Centre for Processable Electronics Imperial College London London SW7 2AZ UK; ^2^ Chu Kochen Honors College Zhejiang University Hangzhou 310058 China; ^3^ Department of Chemistry Molecular Sciences Research Hub Imperial College London London W12 0BZ UK; ^4^ Department of Chemistry University of Manchester Manchester M13 9PL UK; ^5^ Department of Chemistry University of Bath Bath BA2 7AY UK

**Keywords:** bimetallic co‐catalysts, BiVO_4_ photoanodes, density‐functional theory (DFT) calculations, IMPS‐DRT analysis, nickel cobalt phosphides, synergistic effect

## Abstract

Achieving highly performant photoanodes for oxygen evolution is key to developing photoelectrochemical devices for solar water splitting. In this work, BiVO_4_ photoanodes are enhanced with a series of core–shell structured bimetallic nickel‐cobalt phosphides (MPs), and key insights into the role of co‐catalysts are provided. The best BiVO_4_/Ni_1.5_Co_0.5_P and BiVO_4_/Ni_0.5_Co_1.5_P photoanodes achieve a 3.5‐fold increase in photocurrent compared with bare BiVO_4_. It is discovered that this enhanced performance arises from a synergy between work function, catalytic activity, and capacitive ability of the MPs. Distribution of relaxation times analysis reveals that the contact between the MPs, BiVO_4_, and the electrolyte gives rise to three routes for hole injection into the electrolyte, all of which are significantly improved by the presence of a second metal cation in the co‐catalyst. Kinetic studies demonstrate that the significantly improved interfacial charge injection is due to a lower charge‐transfer resistance, enhanced oxygen‐evolution reaction kinetics, and larger surface hole concentrations, providing deeper insights into the carrier dynamics in these photoanode/co‐catalyst systems for their rational design.

## Graphical abstract

Bimetallic metal phosphide co‐catalysts enable a 3.5‐fold enhancement in the photocurrent of BiVO_4_ photoanodes through the synergy of the effective band bending, catalytic activity, and capacitive activity of the co‐catalysts and the hole injection into the electrolyte is significantly improved by the presence of a second metal cation.

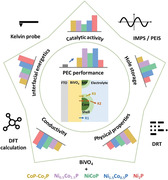

## 1 Introduction

Photoelectrochemical (PEC) water splitting is a promising alternative to traditional hydrogen production from steam reforming of methane.^[^ ^1^ ^]^ For PEC water splitting to reach its full potential, the development of highly efficient, stable photoelectrodes, and especially photoanodes to drive the oxygen‐evolution reaction (OER), is essential. BiVO_4_, an n‐type semiconductor, has attracted significant attention as a photoanode due to its relatively narrow bandgap of ≈ 2.4 eV enabling solar absorption and its deep valence band edge of around +2.5 V versus the reversible hydrogen electrode (V_RHE_) facilitating the OER.^[^ ^2^ ^]^ However, the poor catalytic activity of BiVO_4_ surface cannot fulfill the kinetically demanding OER, resulting in serious charge carrier recombination.^[^ ^3^, ^4^ ^]^ To overcome this problem, various co‐catalysts, normally electrocatalysts such as transition metal phosphates, oxides, and (oxy)hydroxides, have been introduced on BiVO_4_ to boost its catalytic activity.^[^ ^5^ ^]^

Transition metal phosphides (MPs) are well‐known nonprecious Janus catalysts for both the hydrogen and oxygen evolution reactions.^[^ ^6^, ^7^, ^8^, ^9^ ^]^ Compared to pure metal and metal sulfide catalysts, MPs have metallic electronic structures and superior stability, especially under OER conditions. For example, Hu *et al.* demonstrated that nickel phosphide (Ni_2_P) is highly active and stable for the OER,^[^ ^10^ ^]^ and Masa *et al.* demonstrated high OER activity from cobalt phosphide (Co_2_P), both of which are comparable to IrO_2_.^[^ ^6^ ^]^ Interestingly, most reports suggest that MPs undergo surface restructuring during the OER, due to surface oxidation, resulting in core–shell structures comprising a crystalline core and an amorphous oxide layer. These unique core–shell structured electrocatalysts appear to show superior activity compared to the corresponding metal phosphates and (hydro)oxides due to the high conductivity of the crystalline core.^[^ ^6^, ^10^, ^11^ ^]^

Recently, the choice of co‐catalysts for photoanodes has been expanded from mono‐ to bi‐metallic co‐catalysts,^[^ ^12^, ^13^, ^14^ ^]^ and the cooperativity between two metals usually results in improved performance relative to monometallic co‐catalysts. For example, nickel iron (oxy)hydroxide has been extensively studied both as an electrocatalyst for electrolysers and as a co‐catalyst to enhance the performance of photoanodes. Low levels of Fe doping in NiOOH can not only increase the conductivity but also the electrocatalytic activity, up to the Fe solubility limit of 25% above which segregation of FeOOH occurs.^[^ ^15^ ^]^ As a co‐catalyst, the introduction of Fe can prevent photoinduced charging caused by excessive accumulation of holes and thus enhance the performance of photoanodes. Bimetallic phosphides have similarly shown superior electrocatalytic activity in several studies,^[^ ^16^, ^17^, ^18^ ^]^ and also exhibit superior capacitive behaviour.^[^ ^19^, ^20^ ^]^ Although co‐catalysts have been proven to enhance PEC performance, the interactions between the co‐catalysts and semiconductors are poorly understood, and different findings have been reported for the same materials.^[^ ^5^, ^21^, ^22^ ^]^ While these interactions are inherently complex, understanding this complexity is critical to unlocking rapid progress toward efficient photoanodes.

In this work, we have investigated nickel cobalt phosphides (MPs) as co‐catalysts on BiVO_4_ photoanodes and explored the impact of different metal ratios on enhancing the photocurrents by combining multiple structural, physicochemical, computational, and (photo)electrochemical characterization techniques. We discovered that a 3.5‐fold increase in photocurrent can be achieved in BiVO_4_/Ni_1.5_Co_0.5_P and BiVO_4_/Ni_0.5_Co_1.5_P photoanodes compared to BiVO_4_, reaching 3.2 (±0.14) mA cm^−2^ at +1.23 V_RHE_. Kinetic studies confirm enhanced charge transfer in the photoanodes after loading with the bimetallic phosphides, and various processes with different time constants are distinguished by distribution of relaxation times (DRT) analysis. We find that the degree of improvement does not depend only on the catalytic activity of the MPs, but is a synergy of the catalytic activity, effective band bending, and capacitive ability.

## 2 Results and Discussion

### 2.1 Compositions and Morphologies of BiVO_4_ and MPs

**Figure 1:**
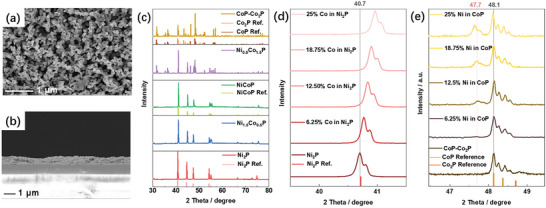
SEM images of the a) top surface and b) cross‐section of BiVO_4_ photoanodes. c) XRD patterns of the prepared metal phosphides: Ni_2_P (red), Ni_1.5_Co_0.5_P (blue), NiCoP (green), Ni_0.5_Co_1.5_P (purple), and CoP‐Co_2_P (brown). A soft curved background was subtracted from the CoP‐Co_2_P sample. d) Shift of the (111) diffraction peak shift in Co‐substituted Ni_2_P. e) Diffraction peaks for the Ni‐substituted CoP and CoP‐Co_2_P samples showing phase separation above 6.25% Ni. Note that all the peaks in the XRD patterns show peak splitting due to K_α1_/K_α2_ diffraction.

BiVO_4_ photoanodes were synthesized following an optimized BiOI‐assisted method that we reported previously.^[^ ^23^, ^24^ ^]^ BiVO_4_ is obtained in the monoclinic crystal system, the most active phase for photocatalysis, as verified by X‐ray diffraction (XRD; JCPDS 00‐014‐0688; Figure S1, Supporting Information).^[^ ^25^ ^]^ We also measured Raman features at 211, 326, 366, 718, and 827 cm^−2^, consistent with clinobisvanite BiVO_4_ (RRUFF ID R070401; Figure S2, Supporting Information). The BiVO_4_ was grown on fluorine‐doped tin oxide (FTO)‐coated glass to ≈700 nm thickness and is porous, as shown in SEM images (**Figure** **1**a,b), with a “worm‐like” morphology and a feature size of ≈200 nm. This morphology provides a large surface area for loading the MP co‐catalysts.

A series of mono‐ and bi‐metallic MPs were synthesized by reducing metal phosphates with various nominal molar ratios of Ni:Co in a 5% H_2_/N_2_ atmosphere at 800 °C. During the reduction, the colored precursor powders turned black, and the successful synthesis of the MPs was verified by XRD (Figure 1c). All diffraction patterns show K_α1_/K_α2_ doublets, indicating a high level of crystallinity in all the samples. Nickel phosphide (Ni_2_P) forms a pure Ni_2_P phase (JCPDS 00‐003‐0953). The cobalt‐doped nickel phosphide Ni_1.5_Co_0.5_P shows diffraction peaks characteristic of Ni_2_P but shifted to higher angles. Similar XRD patterns are seen for a series of samples with increasing Co content, confirming the successful formation of solid solutions (Figure 1d and Figure S3a, Supporting Information). The shift to higher angles indicates lattice contraction and shorter bond lengths, in agreement with the larger difference in electronegativity between Co and P [Co – 1.88, Ni – 1.91, and P – 2.19].^[^ ^26^ ^]^ Nickel cobalt phosphide (NiCoP) is reported to adopt a hexagonal structure (JCPDS 01‐071‐2336), which also has a shifted Ni_2_P diffraction pattern, indicating that Co is soluble up to at least 50% in Ni_2_P.

In contrast to Ni_2_P, Ni_1.5_Co_0.5_P and NiCoP, the monometallic cobalt phosphide sample is a physical mixture of CoP and Co_2_P (JCPDS 03‐065‐2593 and 00‐054‐0413). We therefore refer to this sample as CoP‐Co_2_P. The cobalt‐rich mixed‐metal phosphide sample (named Ni_0.5_Co_1.5_P based on the nominal composition from the reactants) shows characteristic diffraction peaks of both the CoP and Ni_2_P structures (Figure 1c and Figure S3a, Supporting Information), indicating that Ni is poorly soluble in CoP. Further study shows that the characteristic Ni_2_P peaks at 2θ = 47.7°, 54.6°, and 55.5° are clearly detectable above 6.25% Ni (Figure 1e and Figure S3b, Supporting Information). These results show that it is more difficult to replace Co by Ni than Ni by Co in the MPs, in accordance with the Co─P bond being stronger than the Ni─P bond.^[^ ^26^ ^]^ Moreover, the Co_2_P phase is not detectable in any of the Ni‐doped cobalt phosphides, which indicates that forming CoP is favored in the presence of excess P. To support our XRD characterization, DFT calculations were performed on Ni_2_P, Co_2_P, the (Ni_1−_ *_x_* Co*_x_*)_2_P alloy and CoP to obtain the free energy changes (Δ*F*) for different phase separations (Table S3, Supporting Information). Positive (i.e. unfavorable) Δ*F* are obtained for the phase separation of Ni_1.5_Co_0.5_P and NiCoP into the monometallic endpoints, confirming that the formation of these solid solutions is feasible. On the other hand, negative (i.e.favorable) Δ*F* are obtained for the phase separation of Ni_0.5_Co_1.5_P to NiCoP + CoP and for the conversion of Co_2_P to CoP, both in the presence of excess P, which is consistent with our observation of mixed phases in the Co‐rich samples.

**Figure 2:**
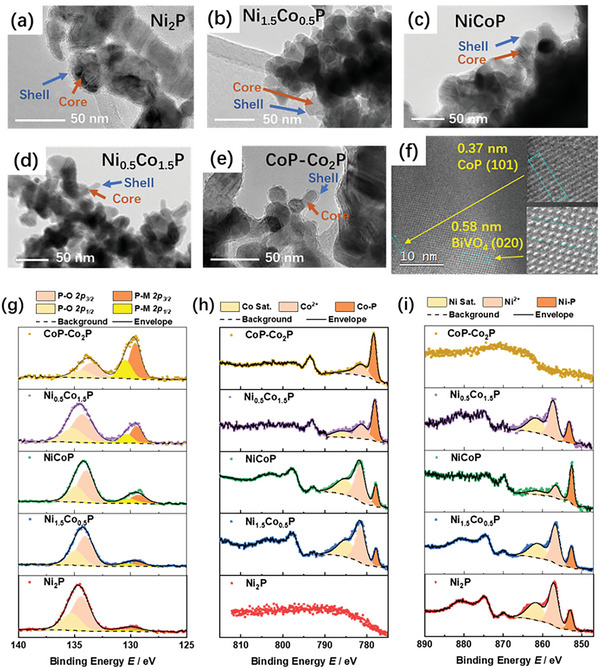
TEM images of the as‐prepared metal phosphides: a) Ni_2_P, b) Ni_1.5_Co_0.5_P, c) NiCoP, d) Ni_0.5_Co_1.5_P, and e) CoP‐Co_2_P. f) HRTEM image of a BiVO_4_/CoP‐Co_2_P photoanode showing the lattice spacing (BiVO_4_: JCPDS 00‐014‐0688; CoP: JCPDS 03‐065‐2593). Enlarged images are provided in Figure S4 (Supporting Information). g–i) XPS spectra of the MPs: g) P 2p, h) Co 2p, and i) Ni 2p.

Transmission electron microscopy (TEM) images show that the MP samples consist of ≈ 50 nm nanocrystals with a crystalline core and a thin amorphous shell (Figure **2**a–e). X‐ray photoelectron spectroscopy (XPS) was used to characterize the surfaces after storage under ambient conditions for around 24 h. Surface oxidation was found to occur readily in all the samples, as evidenced by the presence of oxygen peaks (Figure S5, Supporting Information), which is consistent with previous reports.^[^ ^6^, ^27^, ^28^, ^29^ ^]^

High‐resolution P 2p XPS spectra show two peaks at 129.7 and 133.9 eV, assigned to phosphide and phosphate signals, respectively (Figure 2g). High‐resolution Co 2p XPS spectra show peaks at 778.8 and 781.5 eV, assigned to Co 2p_3/2_ in Co─P and Co─O, respectively, and a doublet at ≈787.5 and 804.8 eV assigned to a combination of satellite features from Co─O and Co─P (e.g., Co─O has satellite features at ≈786 and 803 eV) (Figure 2h). Similarly, deconvolution of the high‐resolution Ni 2p XPS spectra gives peaks that can be assigned to Ni 2p_3/2_ from Ni─P (853.1 eV) and Ni─O (856.8 eV) and satellite peaks at 861.7 eV (Figure 2i). Taken together, the XPS measurements confirm the successful formation of Co and/or Ni phosphides. The presence of oxide‐related features in the XPS measurements and the absence of such features in the XRD measurements indicate that the amorphous shell observed in the TEM images is due to surface oxidation. High‐resolution XPS depth‐profiling spectra in the Ni/Co 2p, O 1s, and P 2p regions using argon ion sputtering provide further evidence for the core–shell structure: for longer sputter times (larger depths), the intensities of the Ni─P (or Co─P) and phosphide peaks increase, while those of Ni^2+^ (or Co^2+^) and O 1s, all three of which are indicative of oxides, decrease (Figure S6, Supporting Information).

**Figure 3:**
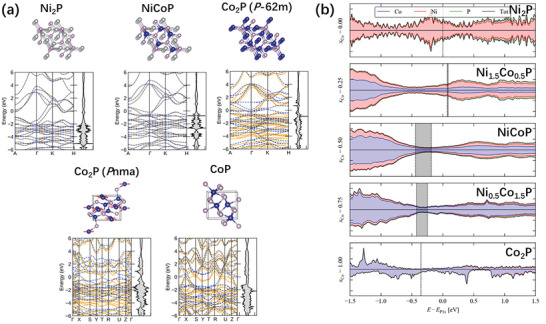
a) Optimized crystal structures and electronic band structures of the metal phosphides obtained from density functional theory calculations. The magnetic moments on the atoms in the Co_2_P and CoP structures are indicated by arrows, but some are smaller than the atom sizes and are therefore not visible. b) Electronic density of states for the (Ni_1−_ *_x_* Co*_x_*)P alloy models (*x* = 0, 0.25, 0.5, 0.75, and 1) predicted from density functional theory calculations. In order to compare between systems the energy is referenced to the average P1s core levels and the zero is set to the calculated Fermi energy *E*_F_ of Ni_2_P (*x* = 0).

Valence‐band XPS spectra were used to determine the valence band maxima. The kinetic energy of the ejected electrons is zero, indicating that the valence bands overlap with the Fermi level and the MPs are conductors (Figure S7a, Supporting Information). This is further evidenced by broad absorption across the UV–vis–NIR region in UV–vis reflectance measurements, which is in marked contrast to the absorption edge typically shown by oxides (Figure S7b, Supporting Information).^[^ ^30^ ^]^ P is less electronegative than O, resulting in more metallic bonding, a higher density of free carriers, and higher conductivity.^[^ ^31^ ^]^ The band structure and electronic density of states (DOS) of some of the MP phases obtained by DFT calculations show overlapping valence and conduction bands and a small density of states around the Fermi level, indicating (semi‐)metallic electronic structures consistent with our measurements (**Figure** **3**a,b). The calculated DoS curves suggest intrinsic carrier concentrations in the range of 10^20^–10^21^ cm^−3^ at room temperature (300 K), which is also consistent with conductive behavior (Table S4, Supporting Information).

### 2.2 Photoelectrochemical Performance of BiVO_4_/MPs Photoanodes

The prepared MPs were drop‐cast on BiVO_4_ photoanodes with a loading of ≈ 0.01 mg cm^−2^ before annealing in an N_2_ atmosphere at 280 °C. TEM and high‐resolution TEM (HRTEM) images confirm successful loading (Figure 2f and Figure S3, Supporting Information). The detection of P 2p and/or Co 2p features from the MPs and a shift of the Bi 2p and V 2p peaks toward larger binding energies in XPS measurements provide further evidence for successful loading and for contact between the phases (Figure S8, Supporting Information). XRD patterns did not show any changes after loading, which indicates the good dispersion and minute loading needed to avoid parasitic absorption (Figure S1, Supporting Information). Finally, energy‐dispersive X‐ray spectroscopy (EDX) mapping clearly shows a uniform distribution of Co and Ni (Figure S9, Supporting Information).

**Figure 4:**
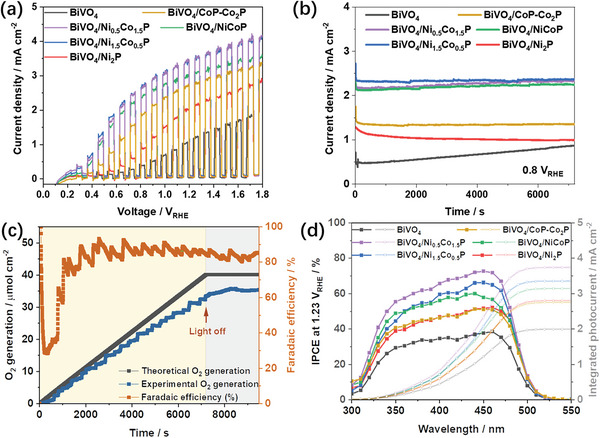
a) Photocurrent density–voltage curves measured for BiVO_4_ and BiVO_4_/metal phosphide photoanodes at a voltage scan rate of 10 mV s^−1^ under chopped simulated sunlight. Reproducibility measurements and statistical analyses are presented in Figure S10 (Supporting Information). b) Photocurrent density stability tests at +0.8 V_RHE_. c) Measured and theoretical oxygen evolution and calculated Faradaic efficiency at +0.8 V_RHE_. d) Incident photon‐to‐current efficiency (IPCE) measurements for the photoanodes at +1.23 V_RHE_ (primary axis) and integrated photocurrents (secondary axis). All measurements were carried out in a 1 m KB buffer solution at pH 9. The photocurrent density‐voltage, stability tests and efficiency measurements were carried out under simulated sunlight (Xe source, AM 1.5G filter, 100 mW cm^−2^), and the IPCE measurements were performed with monochromatic light (Xe source and monochromator). Note that the bare BiVO_4_ photoanode was also annealed in an N_2_ atmosphere at 280 °C, as for the samples loaded with MPs, for comparison.

The PEC performance of the prepared BiVO_4_ photoanodes was evaluated by measuring current–voltage curves under chopped simulated sunlight (Xe source, AM 1.5G filter, 100 mW cm^−2^) in a three‐electrode electrochemical cell, using a 1 m potassium borate buffer electrolyte (KB, pH 9). The photocurrent densities at +1.23 V_RHE_ were measured as 0.93 ± 0.07, 1.67 ± 0.21, 2.32 ± 0.26, 2.75 ± 0.16, 3.20 ± 0.16, and 3.25 ± 0.12 mA cm^−2^ for bare BiVO_4_, BiVO_4_/Ni_2_P, BiVO_4_/CoP‐Co_2_P, BiVO_4_/NiCoP, BiVO_4_/Ni_1.5_Co_0.5_P, and BiVO_4_/Ni_0.5_Co_1.5_P, respectively (**Figure** **4**a; reproducibility measurements and statistical analyses are provided in in Figure S10, Supporting Information). The photoanodes loaded with bimetallic phosphides show superior performance to those loaded with monometallic phosphides. Their performance was confirmed to be stable from chronoamperometry measurements (Figure 4b and Figure S11, Supporting Information), except for a slight decrease in the performance of the Ni_2_P‐containing photoanode which we discuss below. Following an initial drop, the BiVO_4_ photoanode showed a pronounced increase in photocurrent during the stability test that typically saturated below 1 mA cm^−2^ in longer measurements, which was ascribed to the well‐known photocharging of bare BiVO_4_.^[^ ^24^ ^,61,62]^ The amount of O_2_ generated by the BiVO_4_/Ni_1.5_Co_0.5_P photoanode was measured in a gastight PEC cell using an O_2_ sensor (Figure 4c). The O_2_ evolution rate reached an excellent 19 µmol cm^−2^ h^−1^ at +0.8 V_RHE_, demonstrating highly promising device performance. The average Faradaic efficiency for O_2_ evolution, calculated from the ratio of the measured amount of O_2_ to the theoretical maximum derived from the chronoamperometry curves, is around 88%. Possible reasons for not reaching 100% efficiency may include slight photocorrosion of the BiVO_4_, poor O_2_ product desorption, trapping of bubbles on the cell walls, and/or small amounts of gas loss through the rubber fittings.^[^ ^32^ ^]^

To further investigate the effect of the MP co‐catalysts on the photocurrent generation, the incident photon‐to‐current efficiency (IPCE) was measured at +0.8 V_RHE_ and +1.23 V_RHE_ and at different wavelengths (Figure 4d and Figure S12, Supporting Information). The IPCE curves show an edge starting around 525 nm, assigned to the BiVO_4_ bandgap. This IPCE edge is the same for all the photoanodes, confirming that the MPs do not interfere with the light absorption. However, the co‐catalysts all increase the IPCE values to varying degrees, with the best results obtained for BiVO_4_/Ni_1.5_Co_0.5_P and BiVO_4_/Ni_0.5_Co_1.5_P. The decrease in the IPCE values at short wavelengths is due to light absorption by the glass/FTO and/or to reflection. Integrating the product of the IPCE curves and the photon intensity in the AM 1.5G solar spectrum yields calculated photocurrent densities of 2.00, 2.79, 2.75, 3.14, 3.35, and 3.73 mA cm^−2^ at +1.23 V_RHE_ for bare BiVO_4_, BiVO_4_/Ni_2_P, BiVO_4_/CoP‐Co_2_P, BiVO_4_/NiCoP, BiVO_4_/Ni_1.5_Co_0.5_P, and BiVO_4_/Ni_0.5_Co_1.5_P, respectively (Figure 4d). These values are based on the monochromatic light used for the IPCE measurements and are larger than those obtained under 1 sun of simulated sunlight, especially for bare BiVO_4_, which has the poorest catalytic activity (Figure 4a). This mismatch is ascribed to the lower light intensity used for the IPCE measurements (0.18–0.97 mW cm^−2^ vs 100 mW cm^−2^ under simulated sunlight), which results in better matching between the photon flux and hole consumption, and, consequently, less recombination.

The crystallinity and core–shell structure of the MPs was confirmed to be preserved after OER using SEM, TEM, XPS, and XRD. The SEM images of Ni_1.5_Co_0.5_P deposited on a BiVO_4_ photoanode after a stability test at 0.8 V_RHE_ for 8 h in a 1 m KB buffer electrolyte show no changes in morphology (Figure S13, Supporting Information). The TEM images clearly show the retention of the core–shell structure of Ni_1.5_Co_0.5_P deposited on a BiVO_4_ photoanode after the same stability test (Figure S14, Supporting Information). Due to the necessarily minute amounts of metal phosphide used, Co and P XPS spectra of the BiVO_4_/MPs are not suitable for deconvolution, and the MPs are not detectable by XRD (Figures S8 and S15, Supporting Information). Therefore, Ni_1.5_Co_0.5_P was instead loaded onto FTO‐coated glass (for XRD) and carbon paper (for depth‐profiling XPS) and OER was performed under harsher conditions of +1.8 V_RHE_ for 2 h in a 1 m KOH electrolyte (pH 13.7) to verify the preservation of the core–shell structure. After this test, the XRD pattern of Ni_1.5_Co_0.5_P was still detectable, confirming retention of the crystallinity (Figure S16a, Supporting Information), although the intensities of the Ni_1.5_Co_0.5_P diffraction peaks decreased due to visible detachment from the substrate by the many oxygen bubbles evolved during the test. XPS measurements also support the preservation of the composition and core–shell structure, with measurements on the Ni_1.5_Co_0.5_P sample before and after the OER and before and after 60 s of sputtering yielding practically identical spectra consistent with the core–shell structure discussed above (Figure S16b,c, Supporting Information). The observed stability of these metal phosphide core–shell structures is consistent with previous reports.^[^ ^10^, ^33^ ^]^

### 2.3 Role of the Co‐Catalysts

**Figure 5:**
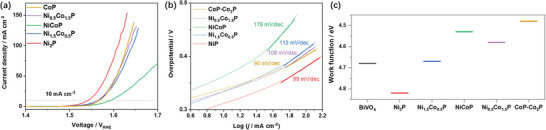
a) Oxygen evolution reaction (OER) activities of metal phosphides (MPs) loaded on a rotating disk electrode (RDE) in a 1 m KOH electrolyte (pH 13.7) at room temperature and pressure, obtained with a voltage scan rate of 10 mV s^−1^ (*iR* corrected). b) Tafel plots for the OER activities of the MPs. c) Work functions of the MPs measured using a Kelvin probe (see Figure S17, Supporting Information).

Electrocatalysts used as co‐catalysts on semiconductors perform a complex set of functions.^[^ ^5^ ^]^ In a bid to establish the origin of the improved photocurrent densities obtained after loading the MPs onto BiVO_4_ photoanodes, the catalytic activity, interfacial energetics, capacitive ability including hole storage, and conductivity were characterized. The OER catalytic activities of the MPs were first characterized using cyclic voltammetry (CV; **Figure** **5**a,b). The Ni_2_P nanoparticles were found to be the most active catalyst, with an overpotential of only 310 mV required to generate a photocurrent density of 10 mA cm^−2^ and a Tafel slope of 99 mV dec^−1^. CoP‐Co_2_P, Ni_0.5_Co_1.5_P, and Ni_1.5_Co_0.5_P have similar activities, with an overpotential of around 330 mV required to generate 10 mA cm^−2^ and Tafel slopes of 90, 108, and 113 mV dec^−1^, respectively. The Tafel slope for CoP‐Co_2_P is lower than that for Ni_2_P, indicating that although the overpotential of CoP‐Co_2_P is larger it can produce current more efficiently in response to changes in applied potential. NiCoP is the least active, requiring more than 360 mV of overpotential to generate a photocurrent density of 10 mA cm^−2^ and having a Tafel slope of 178 mV dec^−1^. The MPs show promising overpotentials and Tafel slopes for the OER, comparable to well‐known IrO_2_ OER catalysts.^[^ ^10^ ^]^ It should be noted that the trends in the overpotentials and Tafel slopes do not mirror the trends in the photocurrents (Figure 4a,b), indicating that the MPs play a more complex role than simply acting as catalysts on the surface of BiVO_4_.

The interfacial energetics were investigated by measuring the work functions of the MPs and BiVO_4_ (i.e., the Fermi levels) using a Kelvin probe (Figure 5c and Figure S17, Supporting Information). Values of 4.82, 4.67, 4.58, 4.53, and 4.48 eV were obtained for Ni_2_P, Ni_1.5_Co_0.5_P, Ni_0.5_Co_1.5_P, NiCoP, and CoP‐Co_2_P, respectively, showing that the work function mostly decreases with Co content. The decreasing trend is consistent with the difference in the work functions of Ni and Co metal [Ni – 5.22 and Co – 5.00 eV].^[^ ^34^ ^]^ The work function of BiVO_4_ is 4.68 eV, which is smaller than that of Ni_2_P but larger than all of the MPs containing Co. Based on these differences, BiVO_4_ should form a Schottky contact with Ni_2_P, which would be beneficial for hole injection, and ohmic contacts with the Co‐containing MPs, which would be detrimental to hole injection (Figure S18, Supporting Information).^[^ ^35^ ^]^ However, the trends in work functions do not follow the trend in the photocurrents and thus cannot explain the superior performance of the BiVO_4_/MP photoanodes and especially the high performance of Ni_1.5_Co_0.5_P and Ni_0.5_Co_1.5_P.

**Figure 6:**
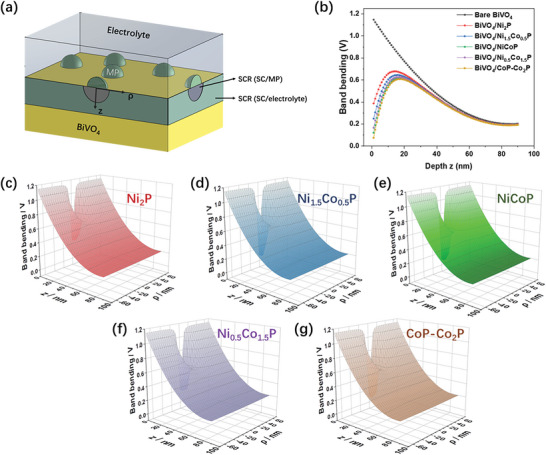
a) Schematic illustration of the space charge regions (SCRs) and depletion regions produced at the interface between BiVO_4_ and the metal‐phosphide (MP) particles and electrolyte. b) Cross‐section of the barrier through the center of the MP island at a radial distance *ρ* =  0  nm, showing the pinched‐off saddle points that are predicted to occur for all of the BiVO_4_/MP photoanodes. c–g) Calculated band bending as a function of radial distance and depth *z* for the five BiVO_4_/MP photoanodes.

Given the small particle size of the MPs, the effective barrier height originating from the “pinch‐off” effect must be considered. This theory describes the modulation of the electric potential behind nanoscale barriers arising from spatial nonuniformities in a semiconductor/metal (SC/M) contact.^[^ ^36^, ^37^ ^]^ Specifically, if the photoelectrode not fully covered by the co‐catalyst and the co‐catalyst particles are small enough, the effective barrier height at the semiconductor/co‐catalyst junction can be much higher than for an ideal SC/M interface, because the large barrier at the semiconductor/electrolyte (SC/E) interface can “pinch it off.” Hence, the majority carriers (i.e., the electrons) do not experience the low barrier height characteristic of a macroscopic SC/M contact, but instead experience the relatively high barrier height imposed by the surrounding SC/E contact (**Figure** **6**).^[^ ^35^, ^38^, ^39^ ^]^ According to an approximate calculation using Tung's pinch‐off model,^[^ ^37^ ^]^ all the samples satisfy the condition for pinch‐off to occur (details of these calculations are provided in Note S1, Supporting Information). The band bending calculated from this model shows potential turbulence within the space charge region (SCR) when a MP island with a 12 nm radius is loaded onto a BiVO_4_ semiconductor (Figure 6c**–**g). For all the photoanodes, the cross‐section of the barrier through the center of the island shows a pinched‐off saddle point. Hence, the pinch‐off effect would balance the low or unfavorable barrier heights for the MPs.

**Figure 7:**
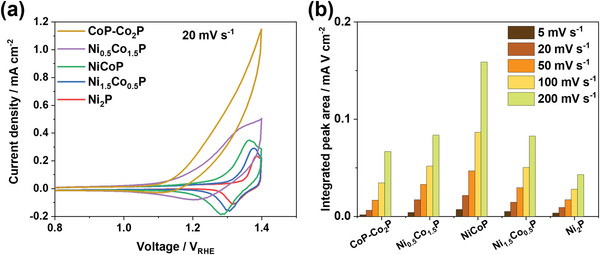
a) Cyclic voltammetry (CV) measurements on the metal phosphides at a scan rate of 20 mV s^−1^. b) Integrated areas of the reduction peaks obtained at various scan rates. All CV measurements were performed in a 1 m KOH electrolyte (pH 13.7).

The capacitive ability of the MPs was investigated using CV measurements. A pair of redox peaks can be observed over the potential window of +1.0 to +1.4 V_RHE_ (**Figure** **7**a). Since the oxidation peaks overlap with the OER reaction, the reduction peaks were chosen for analysis. The redox peaks shift cathodically with increasing Co content, because of the different redox potentials of Co and Ni.^[^ ^40^ ^]^ The peaks for the bimetallic MPs are broader than those for the monometallic ones, and the bimetallic MPs have significantly larger integrated peak areas indicative of higher specific capacitance (Figure 7b). In multimetal compounds, multiple oxidation states involving different metal ions can significantly increase the number of redox sites and greatly improve the capacitive properties.^[^ ^41^ ^]^ The trend in capacitive ability across the MPs partially agrees with the trend in photocurrent densities, which indicates that the capacitive ability is an important factor in the improved performance of the MP‐loaded photoanodes.

To further characterize the capacitive ability, and specifically the hole storage ability, of the MPs, CV scans were performed at different scan rates (Figure S19, Supporting Information). The relationship between the peak current (*i_p_*, mA cm^−2^) and scan rate (ν, mV s^−1^) can be expressed as [Equation (1)]^[^ ^19^, ^42^ ^]^

(1) $$\log\left(i_{p}\right)\;=\;\log\left(a\right)+b\times \log\left(\nu \right)$$

where *a* and *b* are fitting parameters. The *b* value for CoP‐Co_2_P is 1.1, indicative of a rapid surface‐controlled electric double‐layer capacitor, whereas the *b* value for Ni_2_P is 0.56, which is more in keeping with a diffusion‐controlled battery material. Ni_0.5_Co_1.5_P, NiCoP, and Ni_1.5_Co_0.5_P have *b* values of 0.73, 0.81, and 0.65, respectively, spanning the range of 0.5–1 that indicates rapid Faradaic pseudocapacitive behavior. Based on the fitted *b* values, the hole storage mechanism in CoP‐Co_2_P involves double layer charging and discharging, whereas that in Ni_2_P involves bulk redox reactions, and the hole storage mechanisms in Ni_0.5_Co_1.5_P, NiCoP, and Ni_1.5_Co_0.5_P involve surface redox reactions. This supports our previous observation that the crystallinity of the core is maintained, and redox reactions happen in the amorphous shells of all the MPs except for Ni_2_P. This, in turn, explains the photocurrent stability measurements (Figure 4b), where the photocurrent of BiVO_4_/Ni_2_P slightly decreases over time, as this behavior can be attributed to bulk redox reactions in Ni_2_P.

Conductivities were measured using a four‐point probe multimeter and estimated from DFT calculations using semi‐classical Boltzmann transport theory (**Table** **1**). According to the experimental data, the bimetallic catalysts have significantly higher conductivities than the monometallic ones, especially when one of the two metals is present in a higher proportion than the other (i.e., Ni_1.5_Co_0.5_P and Ni_0.5_Co_1.5_P). Some literature assigns the improvements in conductivity to the facile delocalization of electrons over the metal sublattice of the MPs.^[^ ^41^ ^]^ However, we find that the measured conductivities are far smaller than would be expected for metallic conductors (≈10^4^ S cm^−1^), which we attribute to the small particle and feature sizes (e.g., grain boundaries) and to the surface oxidation. The DFT calculations suggest similar, high conductivities for all the bulk MPs, and hence that the differences between the different MP compositions are likely due to the same features that give rise to the lower‐than‐expected conductivities. The conductive nature of the MPs, and the higher conductivities compared to BiVO_4_ (Figure S20, Supporting Information, and Table 1), are likely to contribute to the increased photocurrents.

**Table 1:** Measured and calculated conductivities of the metal phosphides

Metal phosphide	CoP‐Co_2_P	Ni_0.5_Co_1.5_P	NiCoP	Ni_1.5_Co_0.5_P	Ni_2_P	Ni_2_P and CoP‐Co_2_P physical mixtures		
						1:1	3:1	1:3
Conductivity [S cm^−1^] Experiment	0.012 ± 0.002	320.519 ± 79.784	22.854 ± 0.705	212.211 ± 58.230	0.035 ± 0.007	10.714 ± 0.624	2.840 ± 0.057	21.103 ± 1.805
Conductivity [10^4^ S cm^−1^] Simulation	0.83	0.96 ± 0.20	1.28 ± 0.31	3.01 ± 0.05	2.76	N/A	N/A	N/A

Finally, **Table** **2** summarises the properties of the co‐catalysts obtained from the different characterization techniques and their influence on the photoelectrochemical performance of the BiVO_4_/MP photoanodes. It can be concluded from this analysis that the improved photocurrents arise from a synergy between multiple properties and not from just one or two key properties.

**Table 2:** Summary of the properties of the metal‐phosphide (MP) co‐catalysts and an assessment of their impact on the photoelectrochemical (PEC) performance of BiVO_4_/MP photoanodes (red – negative; orange – neutral; green – positive)

		CoP‐Co_2_P	Ni_0.5_Co_1.5_P	NiCoP	Ni_1.5_Co_0.5_P	Ni_2_P
PEC performance	Photocurrent at +1.23 V_RHE_ [mA cm^−2^]	2.72	3.50	3.03	3.50	2.05
Catalytic activity	Overpotential [mV]	330	330	360	330	310
	Tafel slope [mV dec^−1^]	90	108	178	113	99
Interfacial energetics	Fermi level from Kelvin probe/eV to vacuum	−4.48	−4.58	−4.53	−4.67	−4.82
	Effective band bending	Upward	Upward	Upward	Upward	Upward
Hole storage ability	Capacitive ability including hole storage	Low	High	Highest	High	Low
Conductivity	Conductivity (measured) [S cm^−1^]	0.012	320.519	22.854	212.211	0.035
	Conductivity (calculated) [10^4^ S cm^−1^]	0.83	0.96 ± 0.20	1.28 ± 0.31	3.01 ± 0.05	2.76
Physical properties	Oxidation (XPS)	Lowest	Low	Medium	High	Highest
	Particle sizes (TEM)	20.72 ± 6.05	26.47 ± 3.90	20.65 ± 4.59	24.17 ± 4.60	18.12 ± 5.12

**Figure 8:**
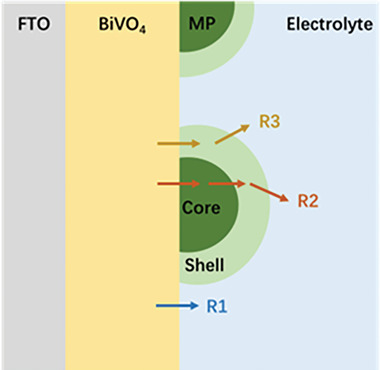
Schematic model showing the possible hole injection routes for bare BiVO_4_ (R1) and BiVO_4_ with metal‐phosphide co‐catalysts (R1‐R3). Note that the relative thicknesses of the FTO, BiVO_4_, and MP layers are for illustration only and are not to scale.

Based on our characterization, we infer that there are several interfaces in the BiVO_4_/core–shell MP/electrolyte structure that lead to different hole injection routes (**Figure** **8**). For bare BiVO_4_ photoanodes, there is only one injection route whereby holes are directly transferred from BiVO_4_ to the electrolyte (R1). By adding the MP co‐catalysts, two more routes are created which are responsible for the enhanced photocurrents: holes are initially drained into the MP core and later stored in the oxidized shell, where reaction with water occurs (R2); and holes are transferred directly to and stored in the shell, where reaction with water occurs (R3). To ascertain their impact on the photoelectrochemical performance, these three routes were examined through a series of kinetic studies.

In R2, the high flux of holes injected into the MP core cannot accumulate in the bulk and instead move toward the core/shell interface. The core/shell interface behaves like and resembles a traditional interface between an electrocatalyst and its metal electrode support. The potential drop is located in the Helmholtz layer or in the amorphous oxides, rather than in the metallic core of the MPs,^[^ ^1^, ^43^ ^]^ and the co‐catalysts cannot store the holes, meaning that they must be consumed efficiently in order to avoid recombination. This route therefore requires high OER catalytic performance, as we summarized in a previous review paper.^[^ ^5^ ^]^

In R3, the semiconductor/shell contact forms a permeable junction. The electrolyte ions surrounding the amorphous co‐catalysts act to locally screen holes, removing the electrostatic potential difference across the interface. The Fermi level of the co‐catalyst coincides with the quasi‐Fermi level of the semiconductor and changes with oxidation state. Hole accumulation in the less active catalysts thus leads to a higher photovoltage, which compensates the poor activity. As we summarized in a recent review paper,^[^ ^1^ ^]^ the permeable junction can therefore accommodate different catalytic activities of the co‐catalysts.^[^ ^38^ ^]^ For this route, the hole storage ability, which is determined by the oxidation states of the shell, is thus much more important than the catalytic activity.

The effective band bending generated by the pinch‐off effect results in a similar hole flux towards the interface in all of the photoanodes (Figure 6). The conductivities of the MPs are all relatively high and can effectively be considered the same (Table 1). For the BiVO_4_/Ni_2_P photoanode, once a high flux of holes reaches the surface of BiVO_4_ the excellent catalytic activity of Ni_2_P boosts the hole injection through the shell (R3) and enhances the photocurrent relative to bare BiVO_4_. However, the enhancement is limited by the inferior capacitive ability of Ni_2_P. Moreover, the battery behavior observed in the capacitance measurements indicates that the core tends to be oxidized during operation, resulting in the lower stability observed in Figure 4b. Progressive oxidation would eventually convert the core–shell Ni_2_P into a fully oxidized amorphous material, at which point hole injection through the core *via* R2 would be impossible. This also accounts for the higher integrated photocurrent from the IPCE measurements compared to that obtained under simulated sunlight (Figure 4d): under the low light intensity in the IPCE measurements, the hole storage is less important due to the reduced hole generation. Moreover, the holes will accumulate in the SCR, leading to surface recombination^[^ ^5^ ^]^ and hence a higher onset potential as observed in Figure 4a. The photocurrent enhancement through the shell *via* R3 in the BiVO_4_/CoP‐Co_2_P photoanode is also limited by this MP co‐catalyst having the lowest capacitive ability. However, CoP‐Co_2_P also shows the highest resistance to oxidation, evidenced by the thinnest oxidation layers determined from XPS and by the electric double‐layer capacitor behavior shown in the capacitance measurements (Figure 2 and Figure S19, Supporting Information). Hence, the BiVO_4_/CoP‐Co_2_P photoanode also benefits from hole injection through the core (R2), leading to a higher and more stable photocurrent than the BiVO_4_/Ni_2_P photoanode.

### 2.4 Charge‐Transfer Kinetics

**Figure 9:**
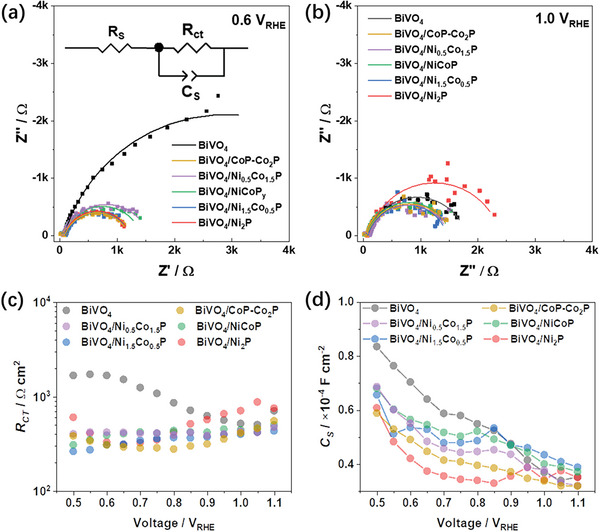
a,b) Photoelectrochemical impedance spectroscopy (PEIS) equivalent circuit and plots for the BiVO_4_ and BiVO_4_/MP photoanodes in a 1 m KB buffer solution (pH 9) under simulated sunlight (Xe source, AM 1.5G, 100 mW cm^−2^) at +0.6 and +1.0 V_RHE_. c,d) Fitted charge‐transfer resistance *R*_ct_ and surface capacitance *C*_s_ as a function of applied bias voltage.

To further understand the impact of the MPs on the interfacial charge transfer, photoelectrochemical impedance spectroscopy (PEIS) measurements were performed under applied biases from +0.5 to +1.1 V_RHE_ using a well‐established equivalent circuit for BiVO_4_ (**Figure** **9**a,b, Supporting Information).^[^ ^44^, ^45^, ^46^, ^47^, ^48^ ^]^ At low biases such as +0.6 V_RHE_, Nyquist plots show smaller second semicircles at low frequencies for the BiVO_4_/MP photoanodes, indicating that the co‐catalysts play an important role in boosting interfacial charge transfer (Figure 9a). At high biases such as +1 V_RHE_, this effect disappears and the BiVO_4_/Ni_2_P photoanode shows an even larger semicircle than bare BiVO_4_ (Figure 9b). This difference can be seen more clearly in the charge transfer resistance values *R*_ct_: the *R*_ct_ of bare BiVO_4_ decreases with increasing applied bias, indicating that applied bias can significantly improve charge separation and injection (Figure 9c), whereas the *R*_ct_ of the majority of the BiVO_4_/MP photoanodes remain low at all biases, indicating that the improved charge separation and injection can be achieved at a lower bias. A notable exception is the BiVO_4_/Ni_2_P photoanode, for which the *R*_ct_ of increases more significantly at biases above +0.8 V_RHE_; this indicates that Ni_2_P blocks charge injection at high bias, perhaps due to bulk oxidation.

The surface capacitance (*C*_s_) of all the photoanodes decreases with increasing bias as a result of the lower depletion capacitances within the SCR (Note S2, Supporting Information). The depletion capacitance is negative due to the built‐in potential (the band bending without applied bias) and the applied bias. At low bias, the MP‐loaded BiVO_4_/MP photoanodes have smaller capacitances compared to the bare BiVO_4_ photoanodes, indicating a more depleted SCR, which may be due to faster OER kinetics. More interestingly, the *C*_s_ of all the photoanodes show small peaks superimposed on the overall declining trends (Figure 9d). These peaks occur at +0.75, +0.95, +0.85, +0.80, +0.85, and +0.80 V_RHE_ for bare BiVO_4_, BiVO_4_/Ni_2_P, BiVO_4_/Ni_1.5_Co_0.5_P, BiVO_4_/NiCoP, BiVO_4_/Ni_0.5_Co_1.5_P, and BiVO_4_/CoP‐Co_2_P, respectively, and can be attributed to activation of catalytic sites. These are most likely oxygen vacancies in bare BiVO_4_^[^ ^24^, ^49^, ^50^ ^]^ and Ni and/or Co in the BiVO_4_/MP photoanodes.

**Figure 10:**
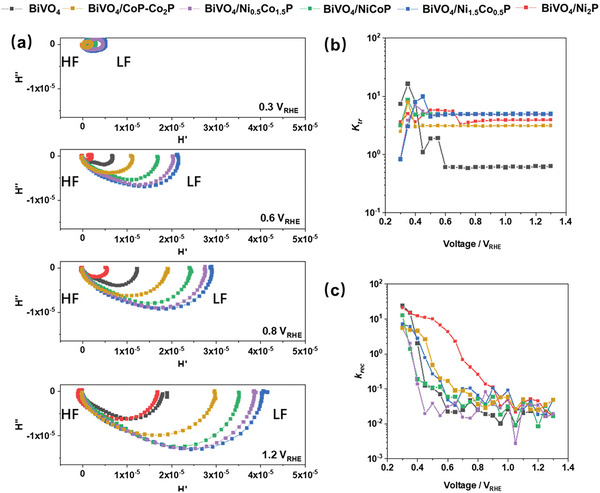
a) Intensity‐modulated photocurrent spectroscopy (IMPS) measurements on BiVO_4_ and BiVO_4_/metal phosphide photoanodes in a 1 m KB buffer solution (pH 9) under different potential biases. b,c) Rate constants *K*_ct_ and *k*_rec_ calculated from the IMPS data.

Intensity‐modulated photocurrent spectroscopy (IMPS) was conducted to investigate the surface kinetics without changing the OER reaction order (Figure S21, Supporting Information). Two complementary methods were used to analyze the IMPS data, namely, the traditional rate‐constant model (RCM) developed by Peter^[^ ^1^ ^]^ and an assumption‐free distribution of relaxation times (DRT) analysis. The RCM method was used to extract kinetic parameters for the charge recombination (*k*_rec_) and charge transfer (*k*_tr_). At a flat band potential of +0.3 V_RHE_, the low‐frequency intercepts are located near the origin, indicating that almost all surface holes recombine, as expected (**Figure** **10**a). When the applied bias is increased the plots change from two semicircles to one semicircle accompanied by increased high‐frequency intercepts, which indicates that biasing the anodes can reduce surface recombination and, therefore, increase the surface hole concentrations, which is confirmed by a reduced *k*_rec_ (Figure 10c).

The MP‐loaded photoanodes have an order of magnitude larger *k*_tr_ than the bare BiVO_4_ photoanodes (Figure 10b), indicating that the MPs contribute significantly to the number of OER active sites.^[^ ^21^ ^]^ On the other hand, the *k*_rec_ of all the photoanodes except for BiVO_4_/Ni_2_P, are similar, suggesting that the MP co‐catalysts do not reduce surface recombination and instead enhance charge transfer. For the BiVO_4_/Ni_2_P photoanode, although *k*_tr_ increases *k*_rec_ is significantly higher than for the other photoanodes, reducing the total charge flow into the electrolyte. The balance between the degree of charge accumulation at the surface, the hole storage ability, and the rate of water oxidation leads to different behavior compared to that when used as a standalone electrocatalyst.^[^ ^51^ ^]^

**Figure 11:**
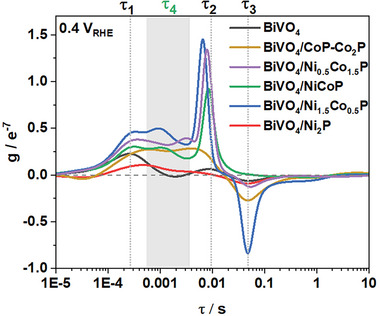
DRT plots for the BiVO_4_ and BiVO_4_/metal phosphide photoanodes.

To distinguish different processes in the BiVO_4_/MP photoanodes, DRT analysis was performed using linear regression with a penalty parameter to prevent overfitting. The validation of the DRT model was first carried out on two sets of simulated IMPS data with three and four pre‐set time constants (τ) (Figure S22, Supporting Information). In the two tests, the DRT model fit the simulated IMPS data and successfully extracted all the pre‐set time constants with an acceptable shift and accurate integrated peak intensities representing the hole flux. This model was subsequently applied to the IMPS data collected for the BiVO_4_/MP photoanodes (**Figure** **11**). Three τ of τ_1_ = 0.27, τ_2_ = 9.5, and τ_3_ = 48 ms were extracted for the bare BiVO_4_ photoanode, whereas an additional τ_4_ = 0.5–5 ms was extracted for the BiVO_4_/MP systems. Based on their signs and magnitudes, the rate constants were assigned to bulk charge carrier transport (τ_1_), hole injection into the electrolyte or MP shells (τ_2_), surface recombination (τ_3_), and hole injection into the MP cores (τ_4_).^[^ ^1^, ^52^, ^53^, ^54^ ^]^

For the MP‐loaded photoanodes, the τ_2_ shift to lower values and the associated peak intensities increase, indicating that the MP co‐catalysts greatly increase the number of active sites for OER and improve the hole injection kinetics (Ni_2_P is again an exception, which we attribute to oxidation). The additional τ_4_ process associated with the BiVO_4_/MP photoanodes is slower than the bulk charge carrier transport because of the barrier at the solid/solid interface, but faster than the hole injection into the electrolyte due to the high conductivities of the MPs. The τ_4_ peak intensity for the BiVO_4_/Ni_2_P photoanode also verifies our previous conclusion that the core of Ni_2_P is easily oxidized, resulting in the loss of the hole injection route through the core.

## 3 Conclusion

In this work, core–shell structured nickel and cobalt metal phosphides with different metal ratios were employed as co‐catalysts on BiVO_4_ photoanodes to enhance the oxygen evolution reaction kinetics. The degree of improvement in the photoelectrochemical performance is not simply related to the catalytic activity of these co‐catalysts, but arises from a synergy of the work function, particle size, catalytic activity, and capacitive ability. The contact between the core–shell structured metal phosphides and BiVO_4_, as well as the contact with the electrolyte, produces various interfaces and leads to three independent routes for hole injection into the electrolyte. The different properties of the co‐catalysts significantly influence the kinetics of these hole injection routes and thereby lead to different degrees of enhancement to the photocurrent density. The best photoanodes were found to be BiVO_4_/Ni_1.5_Co_0.5_P and BiVO_4_/Ni_0.5_Co_1.5_P, due to their excellent hole storage abilities and good catalytic activity, and these systems produce high photocurrent densities of 3.2 (±0.14) mA cm^−2^ at +1.23 V_RHE_. On the other hand, a combination of unfavorable properties, including poor hole storage ability and easy oxidation of the metal phosphide, result in BiVO_4_/Ni_2_P showing limited improvement over bare BiVO_4_ and unstable performance. Kinetic studies using photoelectrochemical impedence spectroscopy and intensity‐modulated photocurrent spectroscopy measurements show that all the BiVO_4_ photoanodes with metal phosphide co‐catalysts display faster charge transfer kinetics and higher surface hole concentrations than bare BiVO_4_ photoanodes. Distribution of relaxation times analysis was used to interpret the IMPS data and to successfully distinguish three or four processes with different time constants. Together, these results clarify the importance of a number of co‐catalyst properties, including catalytic activity, interfacial energetics, and capacitive ability, for the enhancement of photocurrents, and will thereby inform the design of novel photoanodes using bimetallic co‐catalysts to achieve superior photoelectrochemical performance.

## Conflict of Interest

The authors declare no conflict of interest.

## Author Contributions

J.C. and S.E. conceived and planned the experiments. J.C. prepared the materials, assembled the photoanodes, and carried out the tests and characterization. M.D. carried out the Kelvin probe measurements. J.C. and Z.C. designed and carried out the DRT analysis. M.G. carried out the experimental conductivity measurements. J.F., J.S., and S.P. carried out the DFT modeling. J.C. wrote the manuscript with support of S.E., M.D., and J.S. All the authors contributed to analyzing the data, discussing the results and links to literature, and reporting the outcomes.

## Supporting information

Supporting Information

## Data Availability

The data that support the findings of this study are openly available in a research data repository at https://doi.org/10.5281/zenodo.8395647
