# Supplementary material for: BiVO4 Photoanodes Enhanced with Metal Phosphide Co‐Catalysts: Relevant Properties to Boost Photoanode Performance
Source: Small. 2023 Oct 6;20(7):2306757. doi: 10.1002/smll.202306757 (PMC11475583; doi:10.1002/smll.202306757)
Supplement: Supplementary file 1 — Supporting Information [file SMLL-20-2306757-s001.pdf]

NANO · MICRO
small

Supporting Information

for *Small*, DOI 10.1002/smll.202306757

$BiVO_4$ Photoanodes Enhanced with Metal Phosphide Co-Catalysts: Relevant Properties to Boost Photoanode Performance

*Junyi Cui, Matyas Daboczi, Zhenyu Cui, Mengjun Gong, Joseph Flitcroft, Jonathan Skelton, Stephen C. Parker and Salvador Eslava**

Supporting Information

$BiVO_4$ photoanodes enhanced with metal phosphide co-catalysts: relevant properties to boost photoanode performance

*Junyi Cui,[a] Matyas Daboczi,[a] Zhenyu Cui,[b] Mengjun Gong,[c] Joseph Flitcroft,[d] Jonathan Skelton,[d] Stephen C. Parker,[e] and Salvador Eslava[a]**

J. Cui, Dr. M. Daboczi, and Dr. S. Eslava

[a] Department of Chemical Engineering and Centre for Processable Electronics, Imperial College London, SW7 2AZ, London, United Kingdom

E-mail: s.eslava@imperial.ac.uk

Z. Cui

[b] Chu Kochen Honors College, Zhejiang University, 310058, Hangzhou, China

M. Gong

[c] Department of Chemistry, Molecular Sciences Research Hub, Imperial College London, W12 0BZ, London, United Kingdom

Dr. J. Flitcrof, and Dr. J. Skelton

[d] Department of Chemistry, University of Manchester, M13 9PL, Manchester, United Kingdom

Prof. S.Parker

[e] Department of Chemistry, University of Bath, BA2 7AY, Bath, United Kingdom

Synthesis of $BiVO_4$ photoanodes

$BiVO_4$ photoanodes were synthesized following a BiOI-assisted method[1] that we optimized to improve the reproducibility and photoelectrochemical performance. Fluorine-doped tin oxide (FTO)-coated glass (Sigma-Aldrich) substrates were cleaned by ultrasonication in 2% aqueous Hellmanex III solution, deionized water, and isopropyl alcohol, followed by rinsing in deionized water. The FTO surface was ozone treated for 5 min to enhance its surface energy. A 50 mL aqueous plating solution containing 15 mM $Bi(NO_3)_3 \cdot 5H_2O$ (Sigma-Aldrich, 98%), 400 mM KI (Sigma-Aldrich, 99%) and 30 mM lactic acid (Sigma-Aldrich, 88-92%) was prepared, and the pH adjusted to 1.8 using HNO_3 (Sigma-Aldrich, 70%). A 20 mL solution containing 46 mmol p-benzoquinone (Sigma-Aldrich, 98%) in ethanol (VWR, ≥99.8%) was then added slowly to the plating solution. The mixed solution was stirred for 5 min and readjusted to a pH of 3.4 ± 0.05 using HNO_3 (Aldrich, 70%). The resulting solution was used to electrodeposit a BiOI layer on the cleaned FTO-coated glass substrates. The electrodeposition was carried out at a potential of –0.35 $V_{Ag/AgCl}$ for 20 s, followed by a second step at a potential of –0.10 $V_{Ag/AgCl}$ for 17 min (0.37 C cm^{-2}). To transform the BiOI film into $BiVO_4$, it was first covered with 50 µL cm^{-2} of a 200 mM vanadyl acetylacetonate (Sigma-Aldrich, 98%) solution in dimethylsulfoxide (DMSO, Sigma-Aldrich, ≥99.9%), followed by thermal treatment in air at 450 °C for 120 min in a tube furnace (ramp rate 2 °C min^{-1}). Excess of V_2O_5 was eliminated by immersing the film in a stirred aqueous solution of 1M NaOH (Sigma-Aldrich, ≥98%) for 30 min, followed by rinsing with deionized water and drying with a gentle air stream. The $BiVO_4$ photoanode preparation was completed by annealing in N_2 gas (research grade, BOC) at 350 °C for 2 h in a tube furnace (ramp rate 5 °C min^{-1}). For this procedure, the $BiVO_4$ photoanode was mounted at an angle of 30°, facing the N_2 flow.

Synthesis of metal phosphides

Metal phosphides were synthesized by reducing metal phosphates under a H_2/N_2 atmosphere. A total of 1.6 g of metal salts of $CoCl_2$ (Sigma-Aldrich, 99%) and/or $NiCl_2$ (Sigma-Aldrich, 98%) were dissolved in 50 mL deionized water. Different Co/Ni atomic ratios were used as follows: 1:0, 3:1, 1:1, 1:3, and 0:1. After stirring for 5 min, 0.73 g $(NH_4)_2HPO_4$ (Sigma-Aldrich, ≥98%) was added dropwise under continuous stirring. After 1 h, the suspension was heated on a hot plate overnight at 110 °C. Solid metal phosphate solid powders were collected and heated at 800 °C for 3 h with a heating rate of 5 °C/min in 5% H_2/N_2. The black metal phosphides obtained were washed with deionized water and ethanol and dried in vacuum. Nominal compositions of the products, *viz.* Ni_2P, $Ni_{1.5}Co_{0.5}P$, NiCoP, $Ni_{0.5}Co_{1.5}P$ and CoP-Co_2P, were determined based on the proportions of the metal phosphates in the reaction mixture and X-ray diffraction (XRD) measurements.

Loading of metal phosphides on $BiVO_4$

The metal phosphides were loaded onto $BiVO_4$ photoanodes by drop casting followed by annealing. Suspensions containing 10 mg of the metal phosphide in 50 mL IPA were prepared and sonicated for 1 h to obtain a uniform distribution. 100 µL of each suspension was drop-cast on a $BiVO_4$ photoanode prepared according to the procedure outlined above. For fair comparison, the same amount of pure IPA was drop-cast on a bare $BiVO_4$ photoanode. After drying in air, all the photoanodes, including bare $BiVO_4$, were annealed in N_2 at 280 °C for 30 min.

Characterization

XRD patterns were measured on a Malvern Panalytical XRD X'Pert PRO system. Raman spectra were measured using a Bruker SENTERRA II Confocal Raman Microscope. UV-Vis diffuse reflectance spectra were measured using a Shimadzu UV-3000 equipped with a diffuse reflectance integrating sphere. Field-emission gun scanning electron microscopy (SEM) micrographs were obtained using a LEO Gemini 1525 FEG-SEM and an electron high tension (EHT) of 5 kV. High-resolution TEM (HR-TEM) micrographs were obtained using a JEOL JEM-2100Plus electron microscope. X-ray photoelectron spectroscopy (XPS) measurements were obtained on a Thermo Fisher K-Alpha+ with a monochromated Al Kα X-ray source. Binding energies were referenced to the adventitious carbon signal at 284.8 eV. XPS data were processed using the Avantage software.

Conductivity measurements

The electrical conductivities of the metal phosphides were obtained by measuring the resistance difference between a column with and without a sample loaded. Measurements for each sample were repeated three times to obtain an average. The conductivity of the sample was then calculated as the inverse of the resistivity ρ, obtained from:

$$\rho = \frac{RA}{L}$$

where R is the resistance (Ω) and A and L are the cross-sectional area and length, respectively, of the sample in the column.

Cyclic voltammetry measurements

Cyclic voltammetry (CV) measurements were performed using a rotating disk electrode (OrigaTip, Glassy Carbon Tip ø5) with a Pt counter electrode and an Ag/AgCl reference electrode connected to a Solartron potentiostat. Co-catalyst inks were prepared by dispersing 4 mg of the metal phosphide powders in a 1 mL mixture of deionized water and IPA (1:1 v/v) and ultrasonicating for 30 min. After adding Nafion binder solution (catalysts: Nafion = 1:0.5 wt %), the inks were then ultrasonicated for a further 30 mins. 10 μL of each metal phosphide suspension was drop-cast onto the glassy carbon disk of a rotating disk electrode and allowed to dry. Before recording the CV curves, a cleaning step was carried out by sweeping between –0.6 and +1.8 V_{RHE} for 30 cycles at a scan rate of 50 mV/s. CV curves were then measured between –0.6 and +1.8 V_{RHE} in a 1 M KOH aqueous solution (pH 13.7) with a rotation speed of 1000 rpm.

PEC performance

Photoelectrochemical (PEC) performance was measured using a three-electrode setup, with the photoanodes acting as the working electrode (0.28 cm^2 active surface area) against a Pt counter electrode and a KCl-saturated Ag/AgCl reference electrode (Alvatek, MF-2056). The electrolyte was 1 M potassium tetraborate buffer at pH 9. Sunlight was simulated with a Lot Quantum Design Xe lamp source equipped with an AM 1.5G filter. The light intensity was measured with an International Light Technologies SEL033/U photodetector to be 100 mW cm^{-2}. The potential at the working electrode was controlled using a Compactstat potentiostat (Ivium Technologies) and swept with a scan rate of 10 mV s^{-1}.

PEIS characterization

Photoelectrochemical impedance spectroscopy (PEIS) was conducted at direct current (DC) potentials from +0.5 to +1.1 V_{RHE} at a step of 0.05 V and an alternating current (AC) potential of 10 mV with a frequency range of 10 kHz – 0.01 Hz under simulated sunlight (Xe source, AM 1.5G filter, 100 mW cm^{-2}). A typical equivalent circuit was used to fit the results using the Zview software, comprising two resistances and a constant phase element (CPE): a series resistance, R_s (the resistance of the electrolyte, external circuit, and conductive substrate layer); a charge transfer resistance across the semiconductor–liquid junction, R_{ct}; and an interfacial CPE, C_s.

IMPS characterization

For the IMPS measurements the photogenerated charge carrier density is modulated sinusoidally by a time-dependent incident illumination. As a result, the relationship between the photocurrent response and light intensity, known as the transfer function (H), can be expressed in the complex plane, and the surface kinetics of the photoanodes can be extracted from this function. Measurements were conducted using a ModuLab XM PhotoEchem system (Solartron Analytical) with a 455 nm LED (Thorlab M455L3, 37.5 mW cm^{-2}) at potentials from +0.3 to +1.3 V_{RHE} with a step of 50 mV. At each potential step a modulation of 10% of the light intensity was applied over a frequency range from 0.1 Hz - 10 kHz.

Density-functional theory calculations

Theoretical calculations were performed to investigate the energetics, electronic structure and transport properties of the metal phosphide systems using periodic density-functional theory (DFT) as implemented in the Vienna *Ab initio* Simulation Package (VASP) code.[2]

Calculations were performed on the phases of Ni_2P, Co_2P, CoNiP, CoP and P listed in **Table S1**, with initial structures sourced from the Materials Project (MP) database[3] and the Inorganic Crystal Structure Database (ICSD). The MP has a single entry for Ni_2P in the hexagonal $P\bar{6}2m$ spacegroup (**mp-21167**, equivalent to **ICSD: 646117**). There are two entries for Co_2P, *viz.* a $P\bar{6}2m$ structure (**mp-13446**) and an orthorhombic $Pnma$ structure (**mp-22204**). There are two ordered structures for the intermediate CoNiP system with the same $P\bar{6}2m$ space group as Ni_2P and Co_2P but with different relative arrangements of the metal atoms (**mp-1206541**, **mp-1226037**). Preliminary calculations found the **mp-1206541** structure to be lower in energy by 103 meV per F.U. after optimisation, so this structure was used in the calculations. A single entry was found for CoP in the $Pnma$ spacegroup (**mp-22270**). Finally, three entries for elemental P were found in the ICSD, corresponding to the white ($P\bar{1}$; **ICSD: 68326**), violet ($P2/c$; **ICSD: 29273**) and black P phases ($Cmce$; **ICSD: 25253**). Preliminary calculations found the black P phase to be lower in energy than the white and violet P phases by 236 and 23.2 meV $atom^{-1}$, respectively, so the black P phase was used in the calculations.

Electron exchange and correlation were modelled using the PBEsol functional as a base.[4] A Hubbard U correction with U_{eff} = 3.32 and 6.2 eV was applied to the Co and Ni d states, respectively, using the rotationally-invariant model of Dudarev *et al.*[5] These are the calibrated values used by the Materials Project.[3,6] The DFT-D3 dispersion correction was also added to ensure a good description of the P phases.[7] The ion cores were modelled using projector augmented-wave (PAW) pseudopotentials[8,9] with the Co and Ni 4*s*/3*d* and the P 3*s*/3*p* electrons in the valence region. The valence electronic structures were represented in a plane-wave basis

with a kinetic-energy cut-off of 700 eV and Γ-centered Monkhorst-Pack k-point sampling meshes[10] with the subdivisions shown in **Table S1**. These were chosen based on explicit testing to converge the electronic total energies of all the phases to < 1 meV atom^{-1} and the cell pressures to < 1 kbar (0.1 GPa). The structures were fully optimized with thresholds of 10^{-8} eV and 10^{-2} eV Å^{-1} on the electronic total energy and ionic forces, respectively.

For Ni_2P, Co_2P and CoNiP we considered non-magnetic (NM), ferromagnetic (FM) and antiferromagnetic (AF) orderings obtained by placing initial magnetic moments of M = 0, 1 or ± 1 BM on each of the symmetry-inequivalent sets of metal atoms. For Ni_2P and CoNiP all three initial orderings converged to a non-magnetic configuration. For $P\bar{6}2m$ Co_2P we obtained the distinct ferromagnetic and ferrimagnetic (FiM) configurations shown in **Table S2**, of which the FiM configuration was the lowest in energy by 25.3 meV per F.U. and was therefore used in the calculations. For $Pnma$ Co_2P we obtained the ferromagnetic configuration shown in **Table S2**. Finally, for CoP we considered initial NM and FM orderings, which both converged to the FM configuration shown in **Table S2**.

Table S1. List of Systems examined in this work with the source of the initial structure(s) and k-point sampling meshes.

	**Initial Structure(s)**	**k-point Sampling**
**Ni_2P**	mp-21167, ICSD: 646117	8 × 8 × 13
**CoNiP**	mp-1206541, mp-1226037	8 × 8 × 13
**Co_2P ($P\bar{6}2m$)**	mp-13446	8 × 8 × 13
**Co_2P ($Pnma$)**	mp-22204	6 × 10 × 5
**CoP**	mp-22270	5 × 9 × 5
**P (white, $P\bar{1}$)**	ICSD: 68326	1 × 4 × 1
**P (violet, $P2/c$)**	ICSD: 29273	3 × 2 × 1
**P (black, $Cmce$)**	ICSD: 25253	10 × 3 × 7

Note: The initial structures were sourced from the Materials Project (MP) database[3] or the Inorganic Crystal Structure Database (ICSD). k-point meshes are given as the numbers of subdivisions on Γ-centered Monkhorst-Pack grids.[10]

Table S2. Magnetic configurations and relative energies for the $P\bar{6}2m$ and $Pnma$ phases of Co_2P and for CoP.

	**Co_2P ($P\bar{6}2m$, FM)**	**Co_2P ($P\bar{6}2m$, FiM)**	**Co_2P ($Pnma$)**	**CoP**
**$M_{Co(1)}$ [BM]**	0.40	-0.10	1.20	0.33
**$M_{Co(2)}$ [BM]**	0.40	-0.10	1.20	0.33
**$M_{Co(3)}$ [BM]**	0.40	-0.10	1.20	0.33
**$M_{Co(4)}$ [BM]**	0.99	1.25	1.20	0.33
**$M_{Co(5)}$ [BM]**	0.99	1.25	0.04	-
**$M_{Co(6)}$ [BM]**	0.99	1.25	0.04	-
**$M_{Co(7)}$ [BM]**	-	-	0.04	-
**$M_{Co(8)}$ [BM]**	-	-	0.04	-
**M_{Tot} [BM per F.U.]**	1.89	1.65	1.14	0.32
**ΔE [meV per F.U.]**	25.3	0.0	-	-

Note: For each system, the approximate magnetic moments on the Co atoms are given together with the total net magnetic moment per Co_2P or CoP formula unit. The difference in energy per formula unit for the two magnetic configurations of $P\bar{6}2m$ Co_2P is also shown.

The electronic structures of the M_2P and CoP phases were investigated by performing single-point calculations to determine the electronic band energies on uniform k-point grids with 2× the number of subdivisions along each reciprocal lattice vector (i.e. with 8× the number of points). The atom-projected density of states $g(E)$ (DoS) curves were evaluated using VASP with a small Gaussian smearing with a width σ = 0.01 eV. Band structures were obtained using the AMSET code[11] to estimate the band energies, using Fourier interpolation, along strings of k-points forming a path visiting the high-symmetry k-points in the $P\bar{6}2m$ and $Pnma$ Brillouin zones. The intrinsic hole and electron carrier concentrations n_h = n_e were estimated from the DoS curves as:

$$n = \int_{E_\mathrm{F}}^{\infty} f(E, E_\mathrm{F}) g(E) \mathrm{d}E$$

where E_F is the calculated Fermi energy and $f(E, \mu)$ is the Fermi-Dirac distribution function given by:

$$f(E, \mu) = \frac{1}{\exp[(E - \mu)/k_\mathrm{B}T] + 1}$$

The conductivity was estimated using semi-classical Boltzmann transport model implemented in AMSET[11]with a constant electron relaxation time τ = 10^{-14} s. The use of the constant relaxation-time approximation (CRTA) was necessary because the scattering models implemented in AMSET are not suitable for the (semi-)metallic metal phosphides and gave spurious results when tested. For the transport calculations, the dense k-point meshes used for the electronic-structure calculations were further enhanced by an interpolation factor of 25. The conductivity for both p- and n-type carriers was determined at T = 300 K for carrier concentrations between 10^{20} and 5×10^{21} cm^{-3}, and the conductivity corresponding to the $n_\mathrm{h}/n_\mathrm{e}$ estimated from the DoS found by interpolating the resulting data.

A model of the $(Ni_{1-x}Co_x)_2P$ alloy was generated using the method adopted in our previous work.[13,14] An 18-atom $1 \times 1 \times 2$ supercell of the common $P\bar{6}2m$ cell adopted by Ni_2P, CoNiP and Co_2P was generated and the 12 metal atoms systematically replaced to generate all symmetry-inequivalent configurations between Ni_2P (x = 0) and Co_2P (x = 1). The subset of 99 structures corresponding to the x = 0, 0.25, 0.5, 0.75 and 1 compositions targeted in the experiments were selected and fully optimized. The total energies of the optimized structures were then used to construct a thermodynamic partition function for each composition at the experimental formation temperature $T_\mathrm{F} \approx 1075$ K. These were used to assign the structures in each composition an occurrence probability for averaging calculated properties, and to obtain the Helmholtz free energies F and corresponding (averaged) internal energies $\bar{U}$ and configurational entropies S_conf of each composition. Electronic-structure calculations were performed on each structure to obtain averaged DoS curves, carrier concentrations and conductivities using the method outlined above. For consistency, we performed each set of calculations three times starting from the equivalent initial NM, FM and AF magnetic orderings to those tested for the endpoints and took the configuration with the lowest energy. The k-point sampling used for the endpoints was reduced to $8 \times 8 \times 7$ for the alloy supercell. The DoS and carrier concentrations were evaluated with a denser $16 \times 16 \times 13$ sampling mesh, but the substantial storage requirements for the intermediate wavefunction files meant it was necessary to perform the AMSET transport calculations on the smaller $8 \times 8 \times 7$ mesh.

Distribution of relaxation times (DRT) analysis

The DRT analysis is based on the classic model:

$$Y(\omega) = \int_0^\infty \frac{g(t)}{1 + i\omega t} dt$$

where $g(t)$ is the admittance distribution across time constants, and $Y(\omega)$ is the total admittance given by a linear combination of admittances across all time constants. The aim of DRT analysis is to find the $g(t)$ by deconvoluting the linear combination using appropriate regressions. Here we used a parameter-modified Lasso regression with a penalty parameter to avoid overfitting and underfitting.

Note 1: Calculation of the pinch-off effect[15]

1. Parameters used in the calculation:
   W - width of the SCR
   V_{BB} - potential drop across the SCR
   ε - relative permittivity of the semiconductor (68 for $BiVO_4$)[16,17]
   ε_0 - permittivity in vacuum (8.85×10^{-12} F m^{-1})
   q - charge of an electron (1.6×10^{-19} C)
   N - free carrier density (approx. 1×10^{24} m^{-3} for undoped $BiVO_4$)[17]
   R_0 - radius of the metal phosphide particles (12 nm)
   Δ - band-bending difference between the semiconductor/electrolyte and semiconductor/MP interfaces
   V_{CB-f} - potential difference between the conduction band and Fermi level of bare $BiVO_4$ without band bending (0.2 V)

2. The condition for pinch-off is given by:

$$\frac{\Delta}{V_{BB}} > \frac{2R_0}{W}$$

   where W is given by:

$$W = \left(\frac{2V_{BB}\varepsilon\varepsilon_0}{qN}\right)^{1/2}$$

3. The conduction-band energy (E_{CB}) is calculated according to:

$$E_{CB}(\rho = 0, z) = V_{BB}\left(1 - \frac{z}{W}\right)^2 + V_{CB-f} - \Delta\left[1 - \frac{z}{(z^2 + R_0^2)^{0.5}}\right]$$

$$E_{CB}(\rho, z) = V_{BB}\left(1 - \frac{z}{W}\right)^2 + V_{CB-f} + \Delta\left[\frac{zR_0^2}{(z^2 + \rho^2)^{1.5}}\right]$$

Note 2: Calculation of the depletion capacitance[18]

The depletion capacitance C_D within the space-charge region was calculated as:

$$C_D = \frac{\varepsilon\varepsilon_0}{W} = \sqrt{\frac{qN\varepsilon\varepsilon_0}{2}}(V_{BB} - V - \frac{2kT}{q})^{-\frac{1}{2}}$$

where the parameters are the same as used for the calculation of the pinch-off effect, and:
V - applied bias (this value is negative because it is a reverse bias)
k - Boltzmann constant (1.38×10^{-23} m^2 kg s^{-2} K^{-1})
T - temperature (K)

Table S3. Calculated reaction free energies ΔF for phase separations based on the energies obtained from density-functional theory calculations.

	**Reaction**	**ΔF [kJ mol^{-1}]**
**$Ni_{1.5}Co_{0.5}P$**	4 $Ni_{1.5}Co_{0.5}P$ → 3 Ni_2P + Co_2P	75.97
	2 $Ni_{1.5}Co_{0.5}P$ → Ni_2P + NiCoP	14.73
**NiCoP**	2 NiCoP* → Ni_2P + Co_2P (*P*-62*m*)	10.46
	2 NiCoP* → Ni_2P + Co_2P (*Pnma*)	7.64
	2 NiCoP → Ni_2P + 2CoP	46.51
**$Ni_{0.5}Co_{1.5}P$**	4 $Ni_{0.5}Co_{1.5}P$ → Ni_2P + 3 Co_2P	64.21
	2 $Ni_{0.5}Co_{1.5}P$ →NiCoP + Co_2P	8.85
	2 $Ni_{0.5}Co_{1.5}P$ + P → NiCoP + 2 CoP	-96.21
**CoP-Co_2P**	Co_2P (*P*-62*m*) → Co_2P (*Pnma*)	-2.82
	Co_2P (*P*-62*m*) + P → 2 CoP	-63.33
	Co_2P (*Pnma*) + P → 2 CoP	-60.51

* NiCoP energies are calculated from the optimized reported crystal structure rather than from the alloy model (i.e. based on a single structure rather than a thermodynamic average of multiple structures).

Table S4. Calculated intrinsic carrier concentrations (n_e for electrons and n_p for holes) and conductivities for the metal phosphides along the x/y directions $(\sigma_{xx} + \sigma_{yy})/2$ and z direction together with the average $\sigma_{ave} = (\sigma_{xx} + \sigma_{yy} + \sigma_{zz})/3$.

	**Ni_2P**	**$Ni_{1.5}Co_{0.5}P$**	**NiCoP**	**$Ni_{0.5}Co_{1.5}P$**	**Co_2P**
**$n_e = n_p$ [10^{20} cm^{-3}]**	7.30	6.16 ± 0.58	3.44 ± 1.04	4.28 ± 0.50	4.85
**$[\sigma_{xx}(n) + \sigma_{yy}(n)]/2$ [10^4 S cm^{-1}]**	3.31	3.88 ± 0.19	1.68 ± 0.49	1.27 ± 0.28	0.94
**σ_{zz} (n) [10^4 S cm^{-1}]**	1.65	1.27 ± 0.15	0.47 ± 0.10	0.33 ± 0.08	0.62
**σ_{ave} (n) [10^4 S cm^{-1}]**	2.76	3.01 ± 0.05	1.28 ± 0.31	0.96 ± 0.20	0.83
**$[\sigma_{xx}(p) + \sigma_{yy}(p)]/2$ [10^4 S cm^{-1}]**	3.41	3.94 ± 0.20	1.59 ± 0.44	1.19 ± 0.25	0.85
**σ_{zz} (p) [10^4 S cm^{-1}]**	1.60	1.26 ± 0.16	0.49 ± 0.16	0.32 ± 0.08	0.62
**σ_{ave} (p) [10^4 S cm^{-1}]**	2.81	3.05 ± 0.05	1.22 ± 0.26	0.90 ± 0.17	0.78

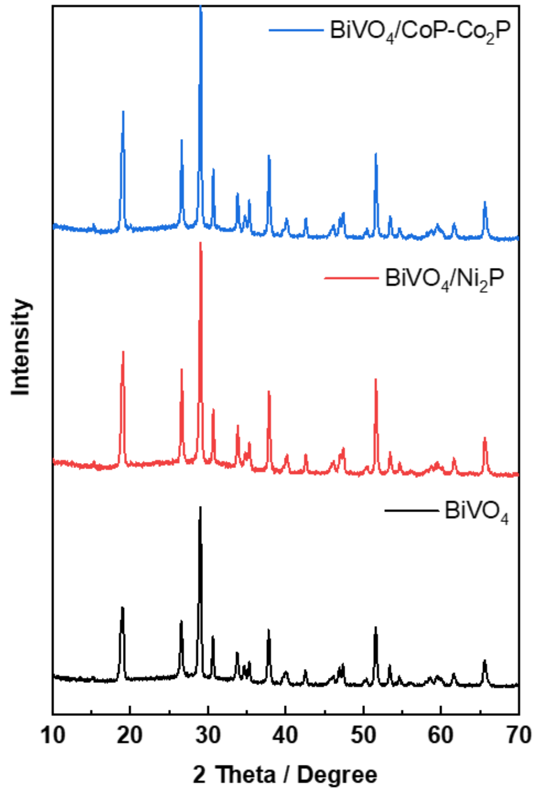

Figure S1. XRD patterns for the photoanodes prepared in this work: $BiVO_4$ (black), $BiVO_4/Ni_2P$ (red), and $BiVO_4/CoP\text{-}Co_2P$ (blue).

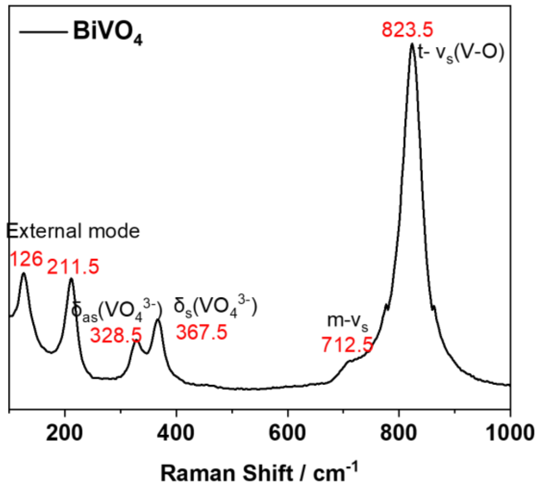

Figure S2. Raman spectrum of the bare $BiVO_4$ photoanodes prepared in this work.

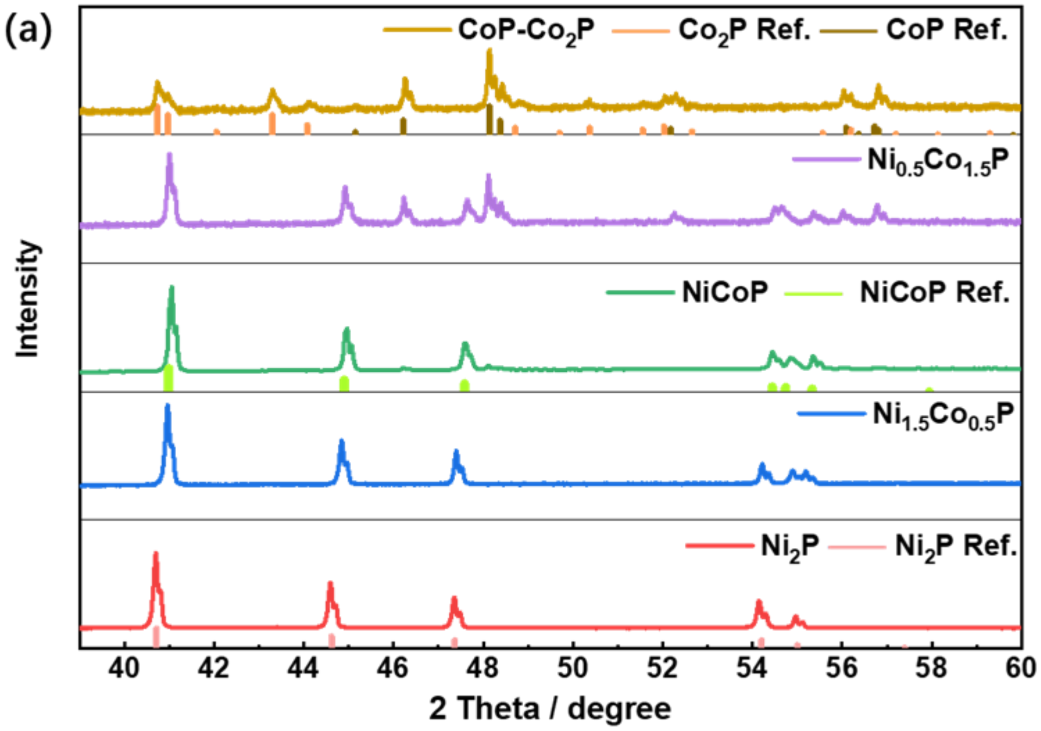

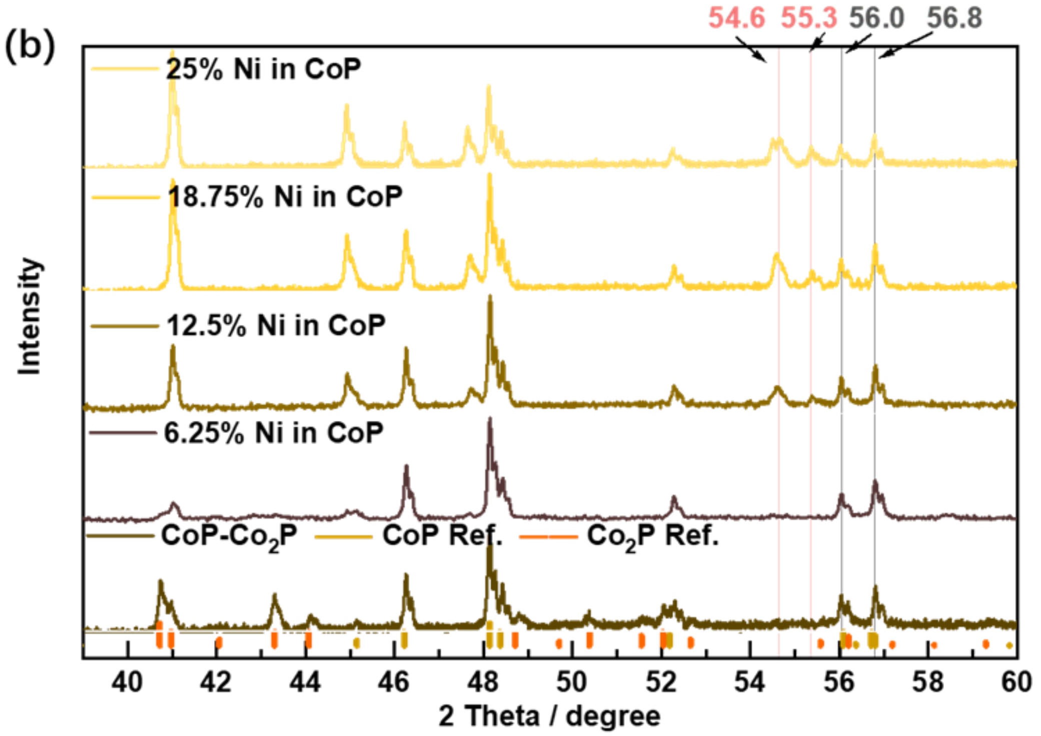

Figure S3. (a) Enlarged XRD patterns for the metal phosphides prepared in this work. (b) Enlarged XRD patterns of Ni-substituted CoP and CoP-Co_2P. Both sets of measurements are compared to reference patterns (Ni_2P - JCPDS 00-003-0953, NiCoP - JCPDS 01-071-2336, CoP - JCPDS 03-065-2593, and Co_2P - JCPDS 00-054-0413).

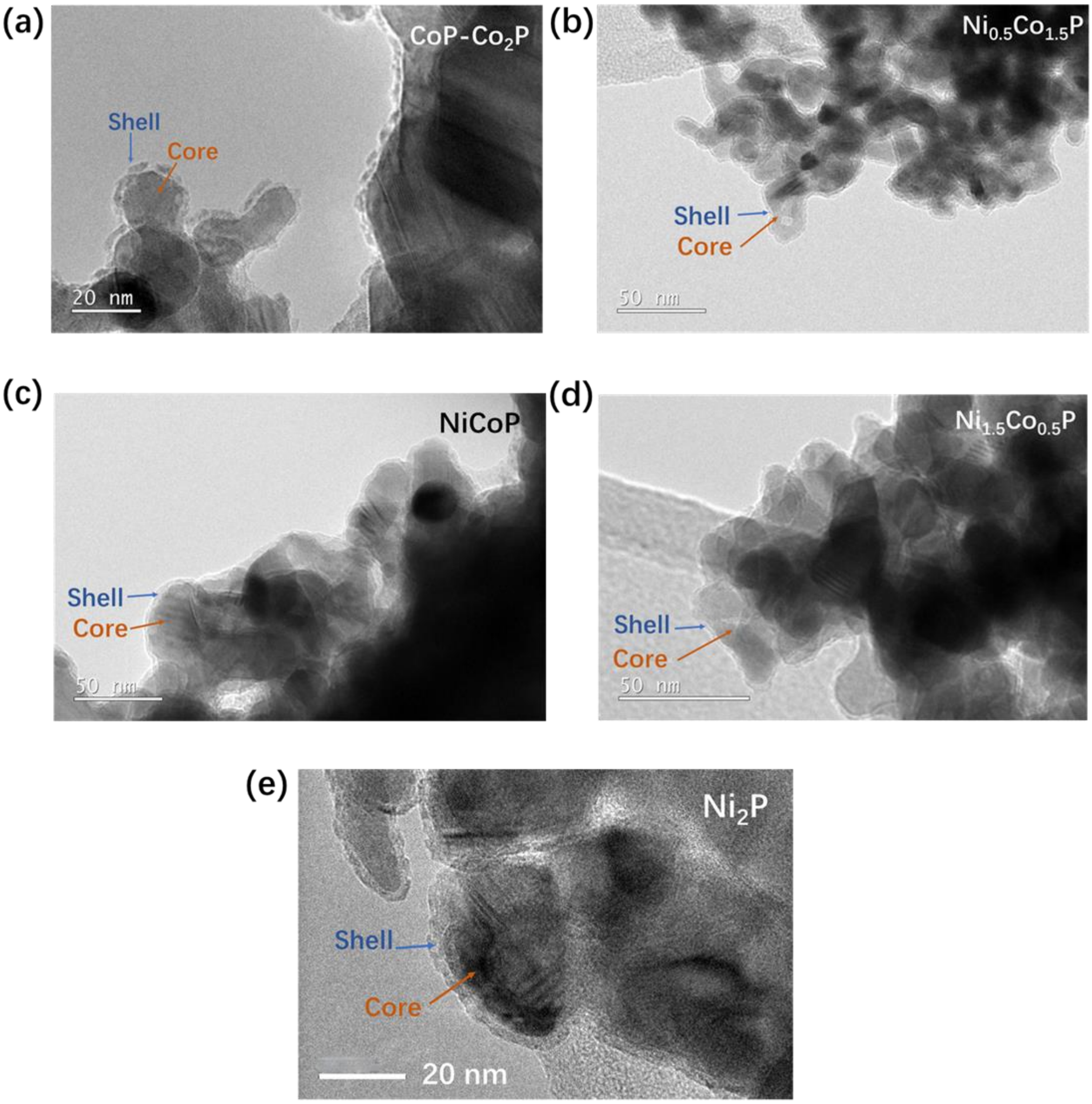

Figure S4. HR-TEM micrographs of metal phosphides for (a) CoP-Co_2P, (b) $Ni_{0.5}Co_{1.5}P$, (c) $NiCoP$, (d) $Ni_{1.5}Co_{0.5}P$, and (e) Ni_2P;

(f)

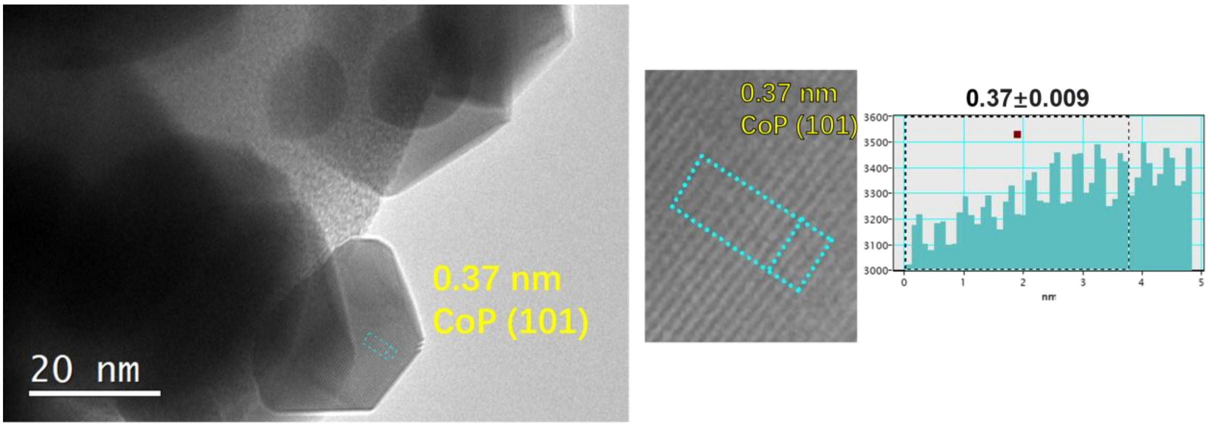

(g)

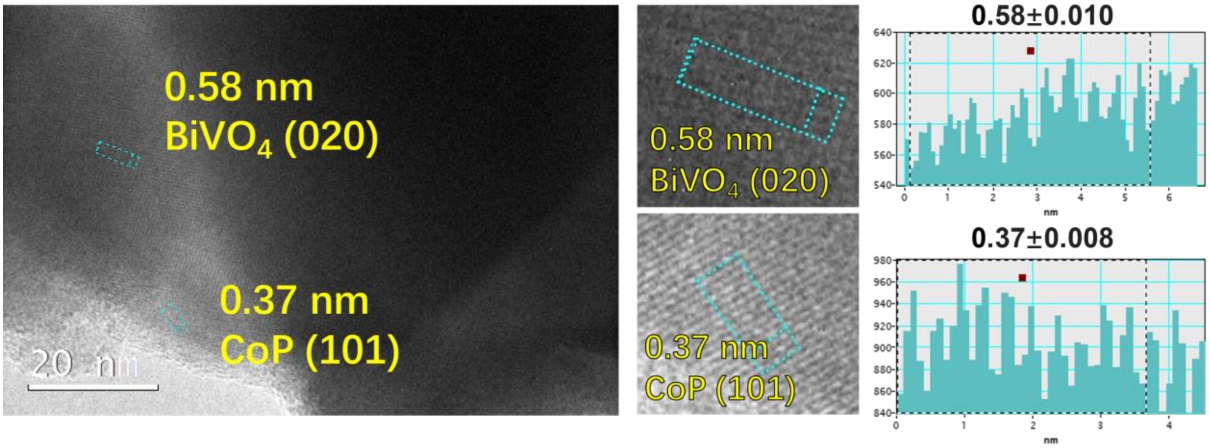

(h)

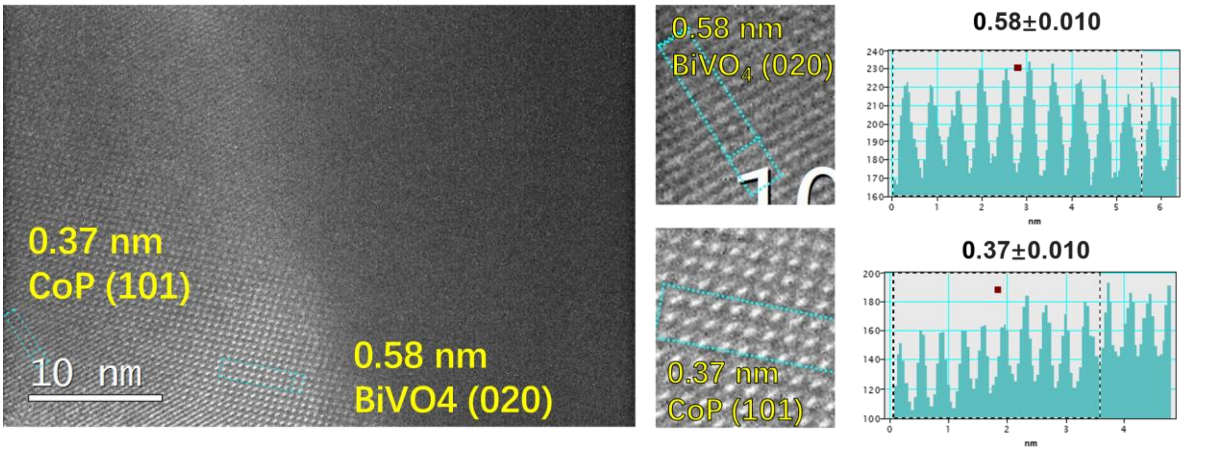

Figure S4 (continued). HR-TEM micrographs and lattice space calculation for (f)/(g) the $BiVO_4$/CoP-Co_2P and (h) $BiVO_4$/$Ni_{0.5}Co_{1.5}P$ photoanodes prepared in this work ($BiVO_4$: JCPDS 00-014-0688; CoP: JCPDS 03-065-2593).

(i)

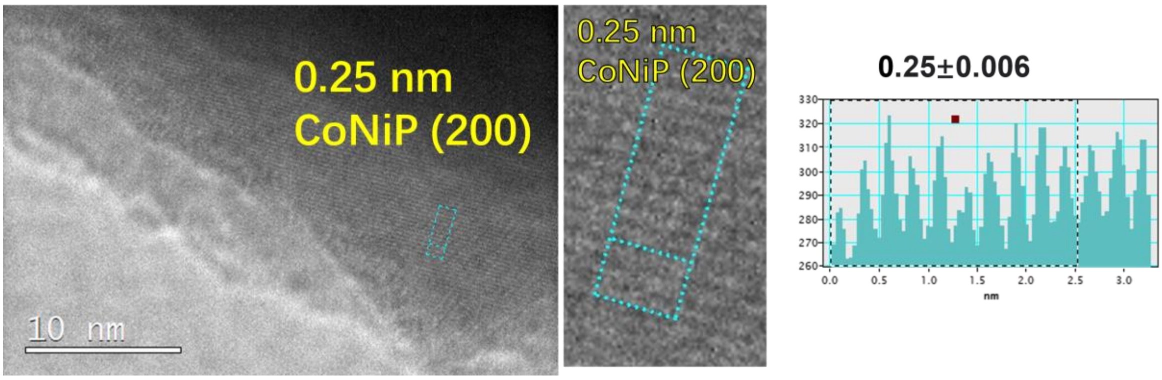

(j)

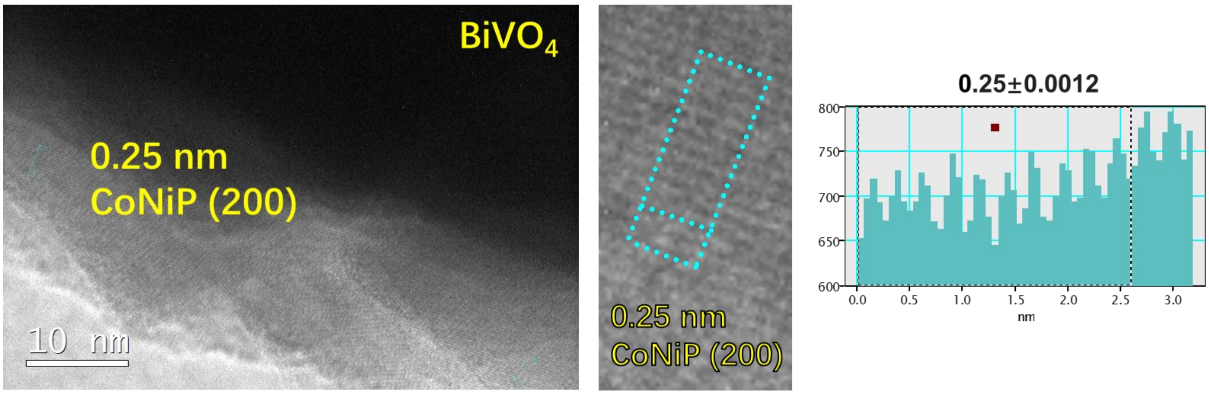

Figure S4 (continued). HR-TEM micrograph and lattice space calculation for (i)/(j) the $BiVO_4$/NiCoP photoanodes prepared in this work ($BiVO_4$: JCPDS 00-014-0688; CoNiP: JCPDS 01-071-2336).

(k)

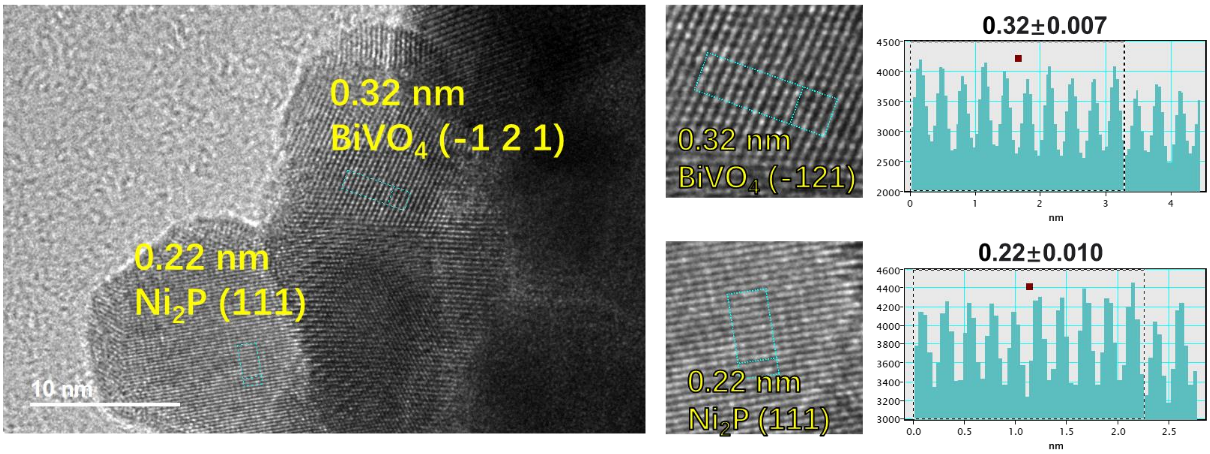

(l)

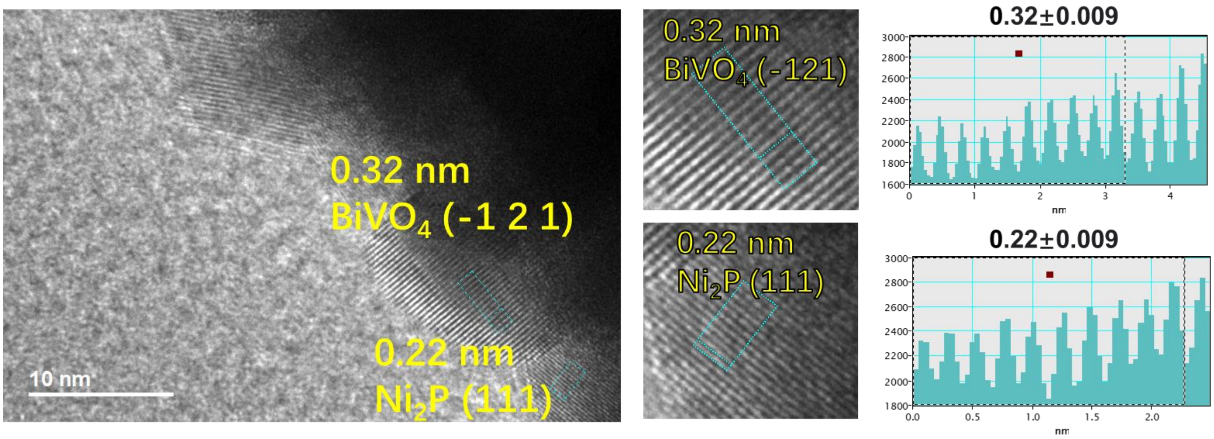

(m)

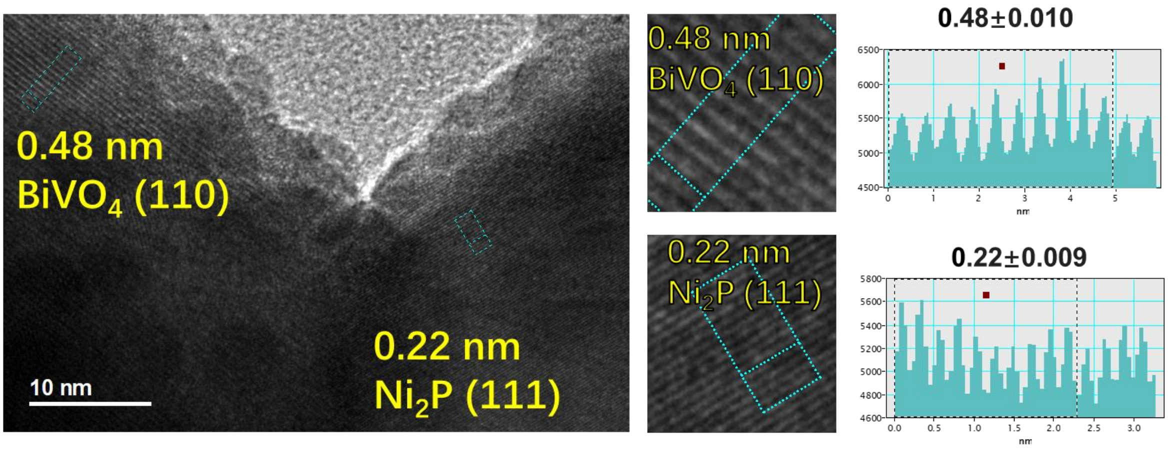

Figure S4 (continued). HR-TEM micrograph and lattice space calculation for the (k)/(l) $BiVO_4/Ni_{1.5}Co_{0.5}P$ and (m) $BiVO_4/Ni_2P$ photoanodes prepared in this work ($BiVO_4$: JCPDS 00-014-0688; Ni_2P: 00-003-0953).

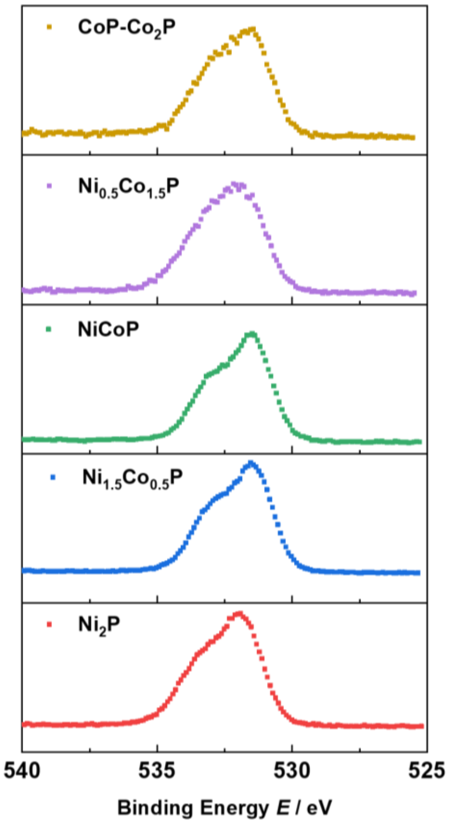

Figure S5. High-resolution O 1*s* XPS spectra of the metal phosphides prepared in this work.

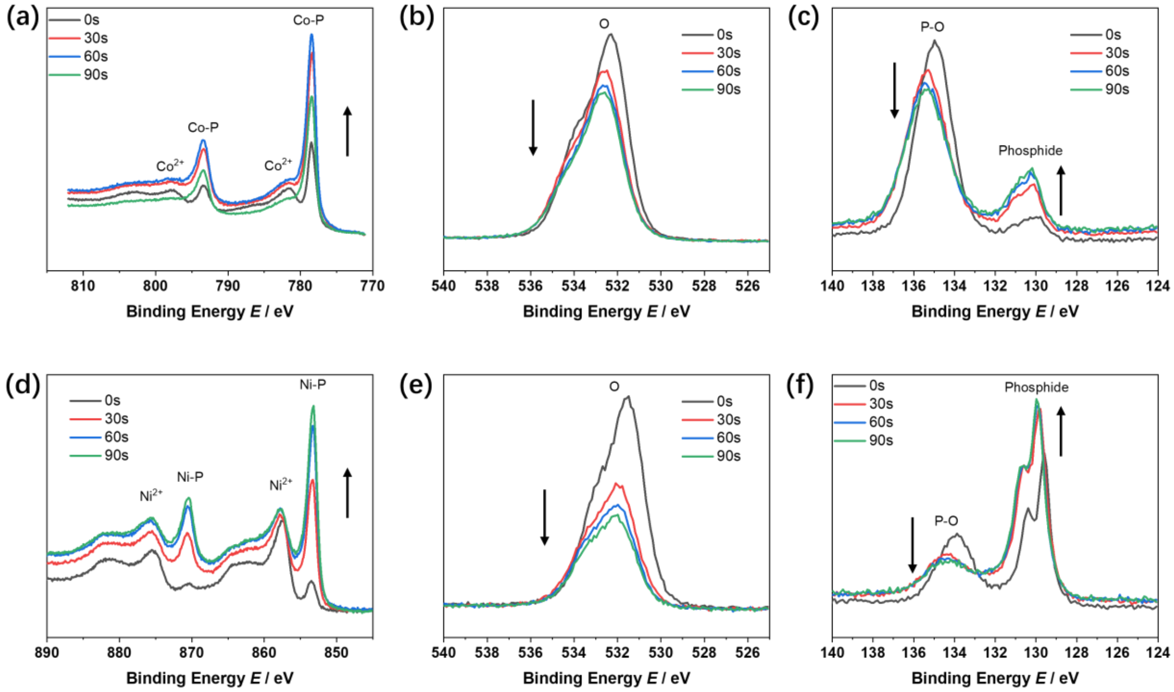

Figure S6. High-resolution depth-profiling XPS spectra of the samples prepared in this work: (a)-(c) Ni 2*p* (a), O 1*s* (b), and P 2*p* (b) of Ni_2P; and (d)-(f) Co 2*p* (d), O 1*s* (e), P 2*p* (f) of CoP-Co_2P.

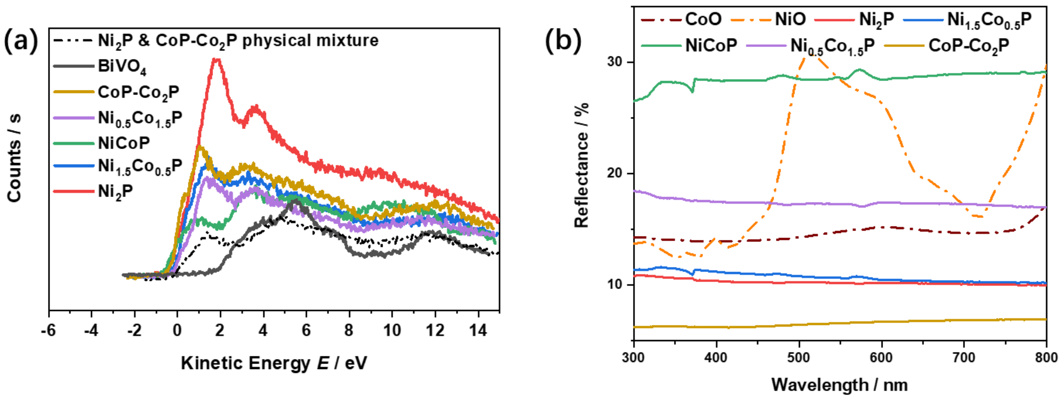

Figure S7. (a) Valence band XPS spectrum of the $BiVO_4$ and metal phosphides prepared in this work. (b) UV-Vis diffuse reflectance spectra of the metal phosphides and NiO.

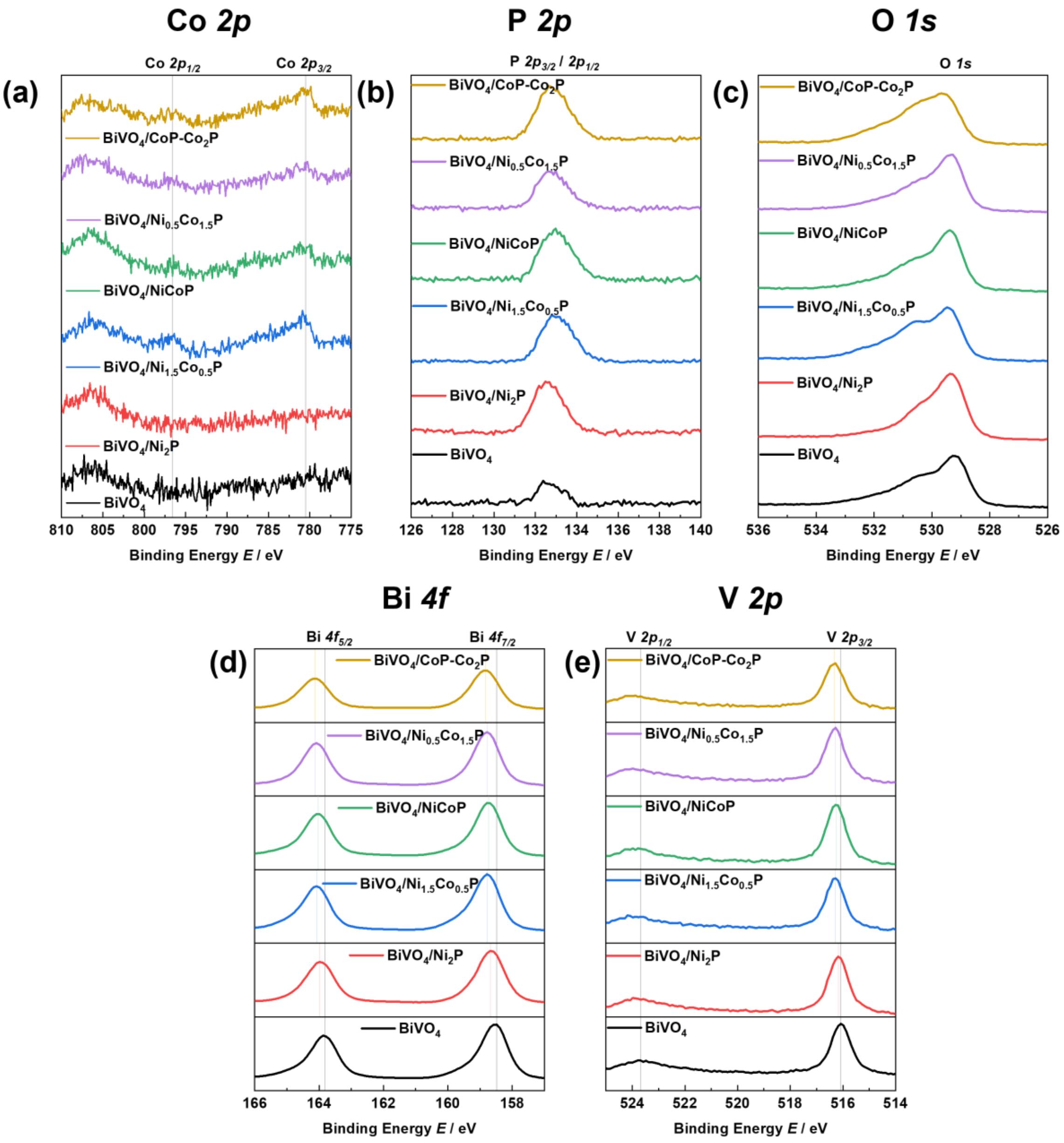

Figure S8. High-resolution XPS spectra of the $BiVO_4$/metal phosphide photoanodes prepared in this work: (a) Co 2*p*; (b) P 2*p*; (c) O 1*s*; (d) Bi 4*f*; and (e) V 2*p*.

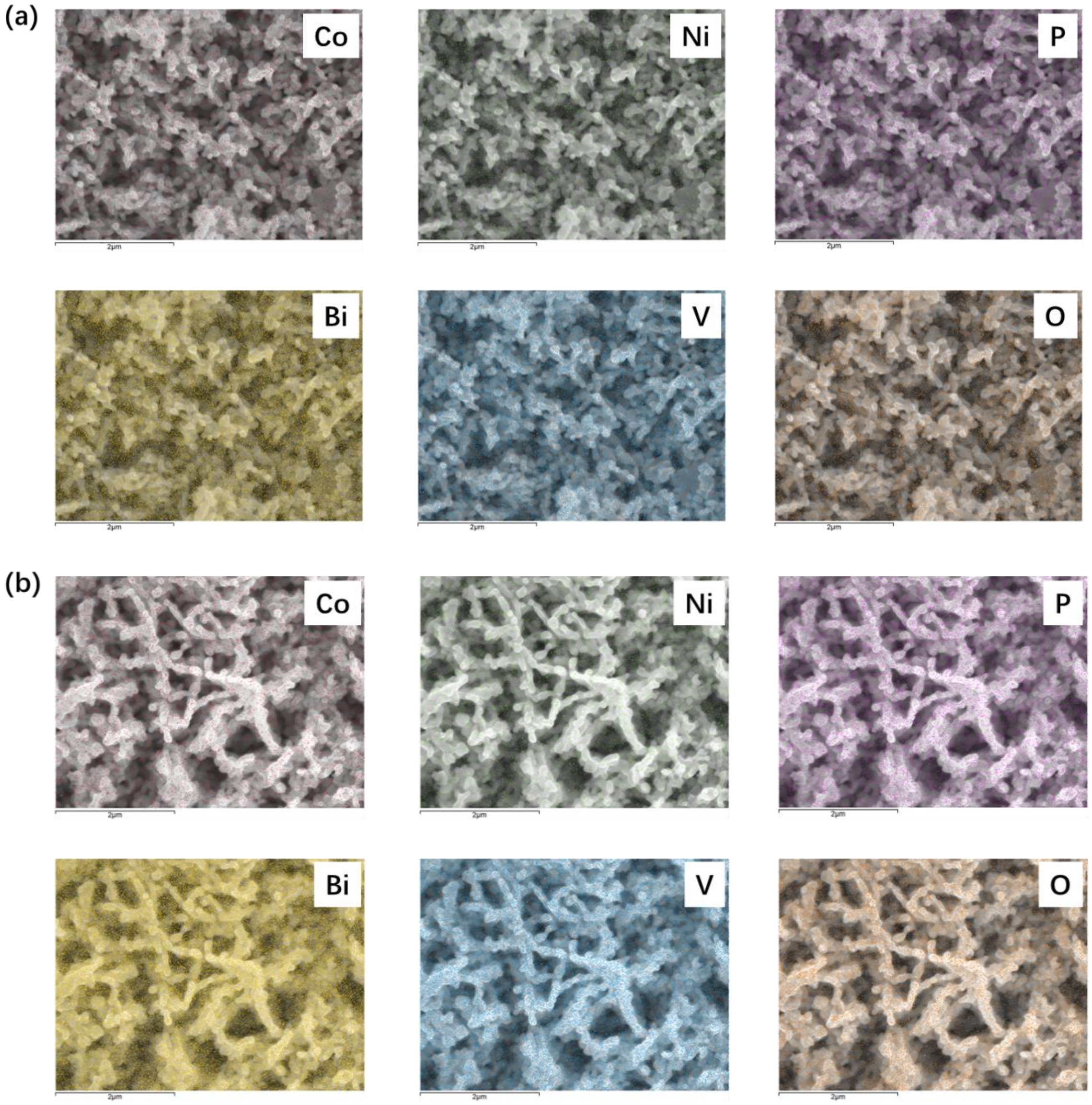

Figure S9. EDX mapping of the distributions of Co (red), Ni (green), P (purple), Bi (yellow), V (blue), and O (orange) in the $BiVO_4/Ni_{1.5}Co_{0.5}P$ (a) and $BiVO_4/Ni_{0.5}Co_{1.5}P$ (b) photoanodes prepared in this work.

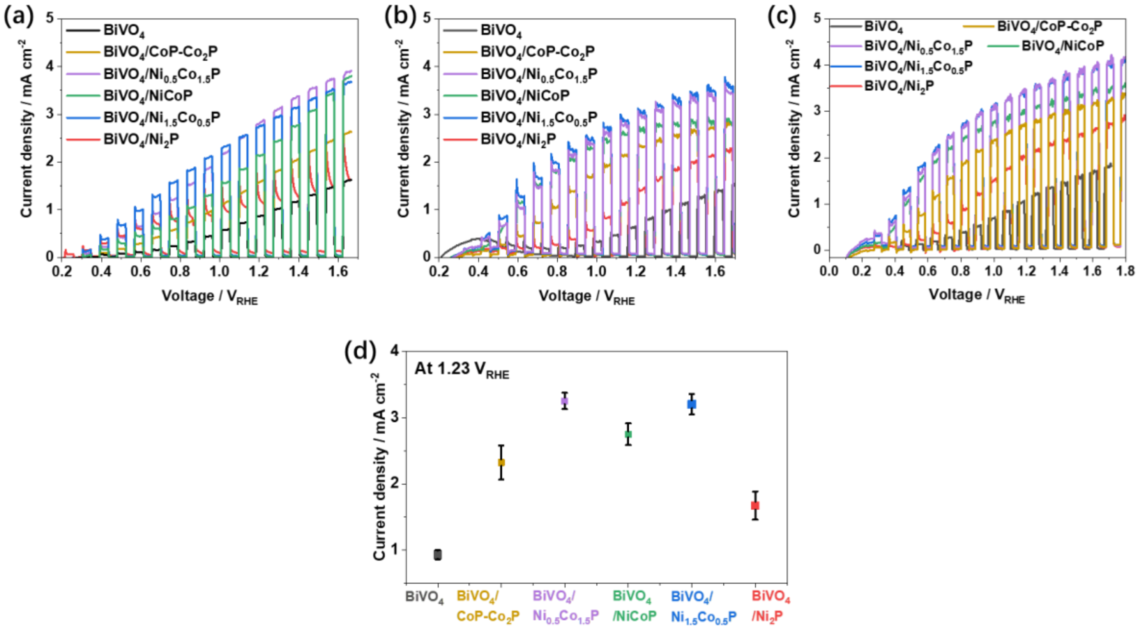

Figure S10. (a)-(c) Reproducibility and (d) error statistical analysis of the photocurrent density-voltage curves collected for the $BiVO_4$ and $BiVO_4$/metal phosphide photoanodes prepared in this work in a 1 M KB electrolyte (pH 9), with a voltage scan rate of 10 mV s^{-1}, under chopped illumination (Xe source, AM 1.5G filter, 100 mW cm^{-2}).

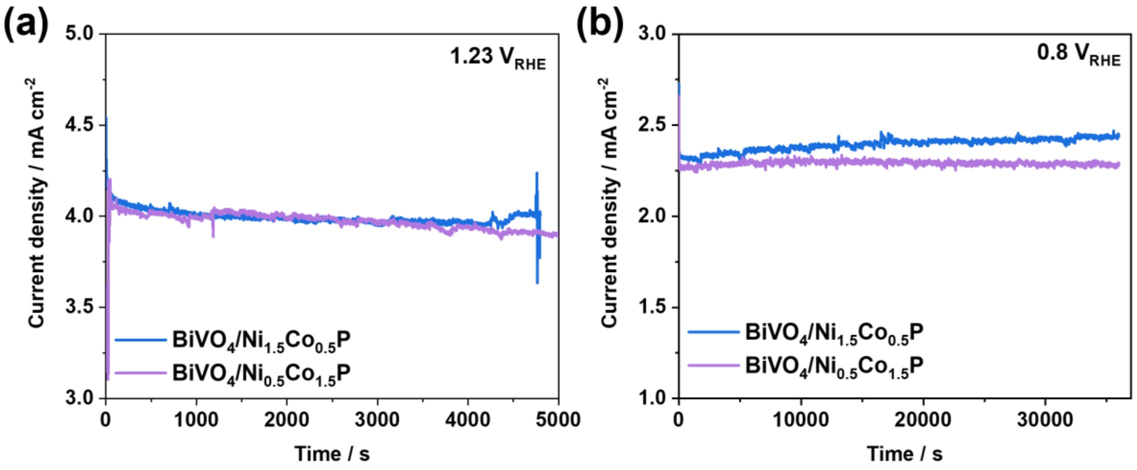

Figure S11. Photocurrent density stability tests on the $BiVO_4/Ni_{1.5}Co_{0.5}P$ and $BiVO_4/Ni_{0.5}Co_{1.5}P$ photoanodes prepared in this work at +1.23 V_{RHE} (a) and +0.8 V_{RHE} (b).

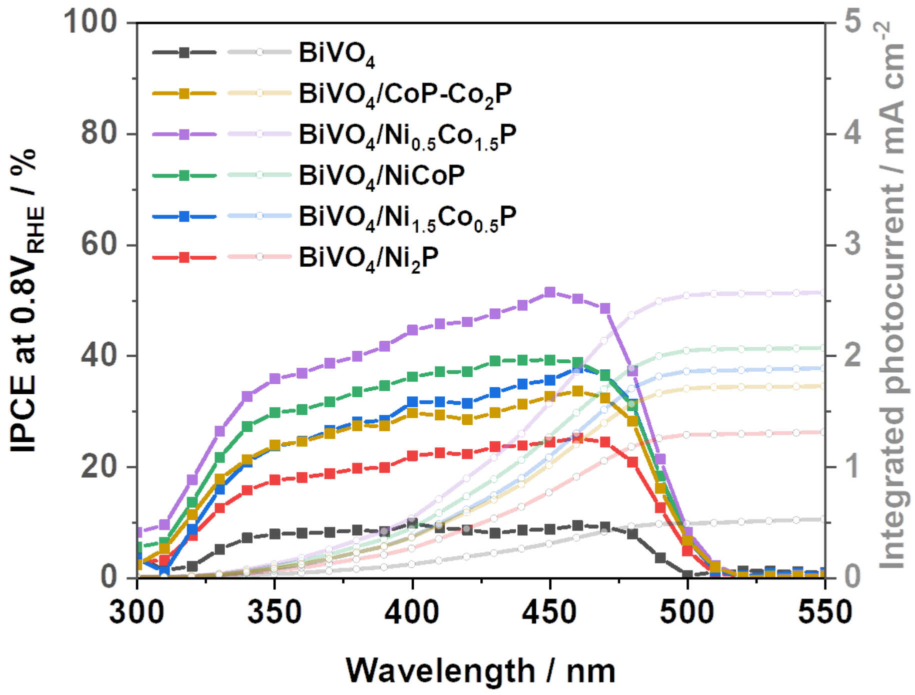

Figure S12. IPCE plots for the $BiVO_4$ and $BiVO_4$/metal phosphide photoanodes prepared in this work at +0.8 V_{RHE} (primary axis) and integrated photocurrents (secondary axis).

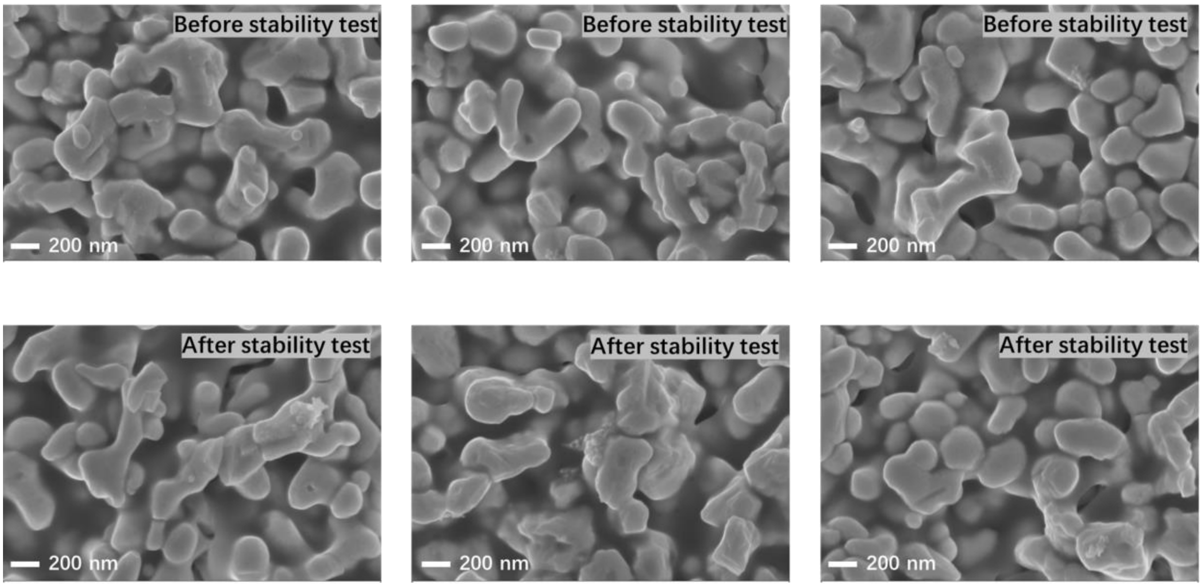

Figure S13. SEM micrographs for the $BiVO_4/Ni_{1.5}Co_{0.5}P$ photoanodes before and after a stability test.

(a)

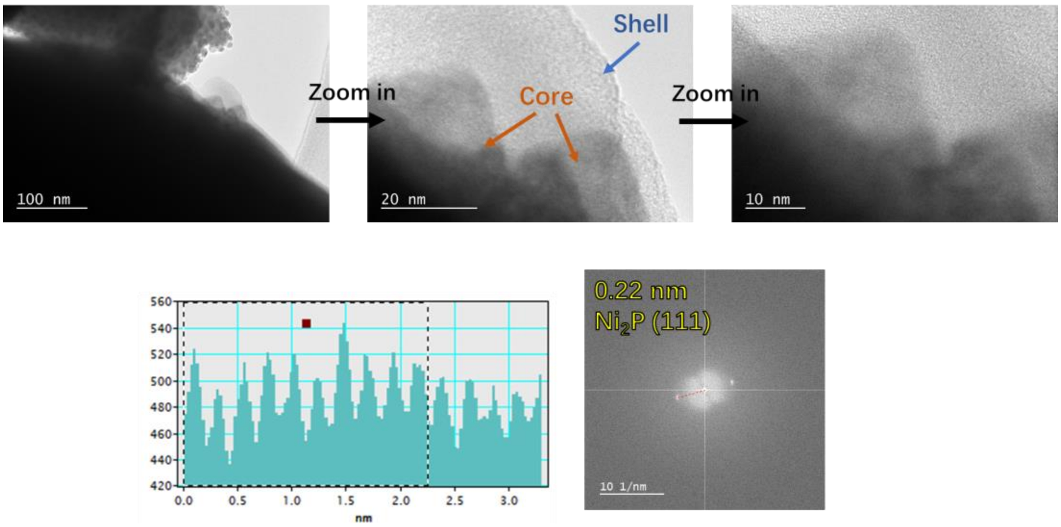

(b)

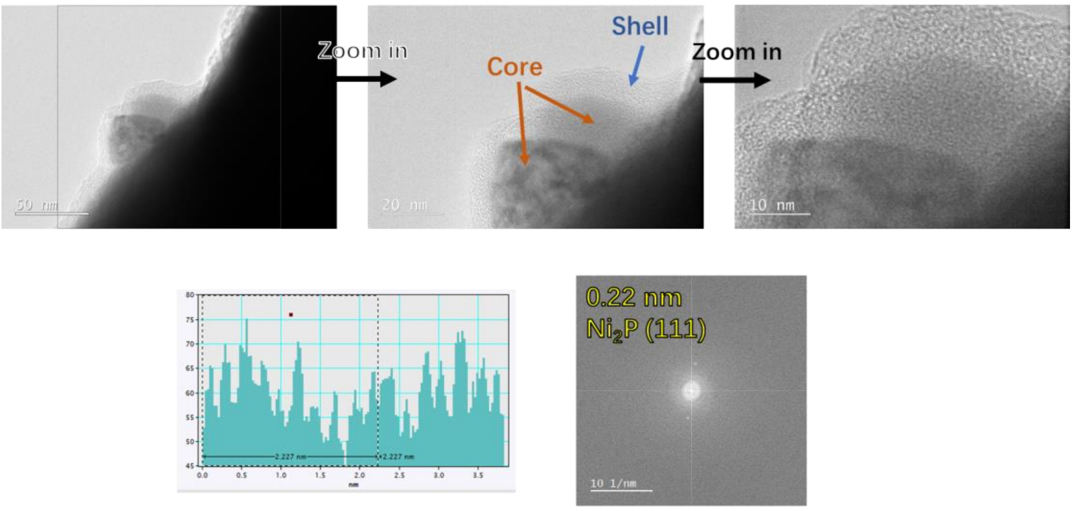

(c)

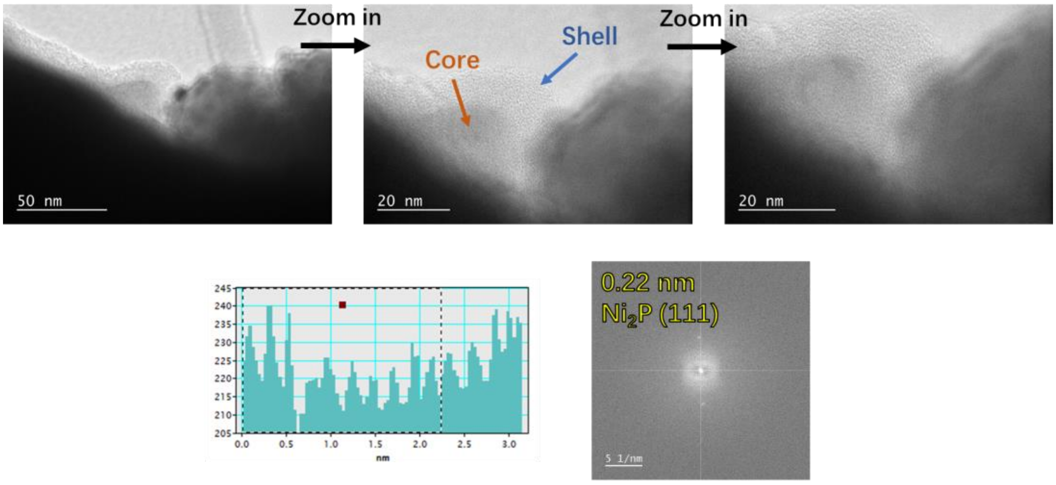

Figure S14. (a-c) HR-TEM micrographs, lattice space calculation, and FFT image for the $BiVO_4/Ni_{1.5}Co_{0.5}P$ photoanodes prepared in this work after a stability test (Ni_2P: 00-003-0953).

(d)

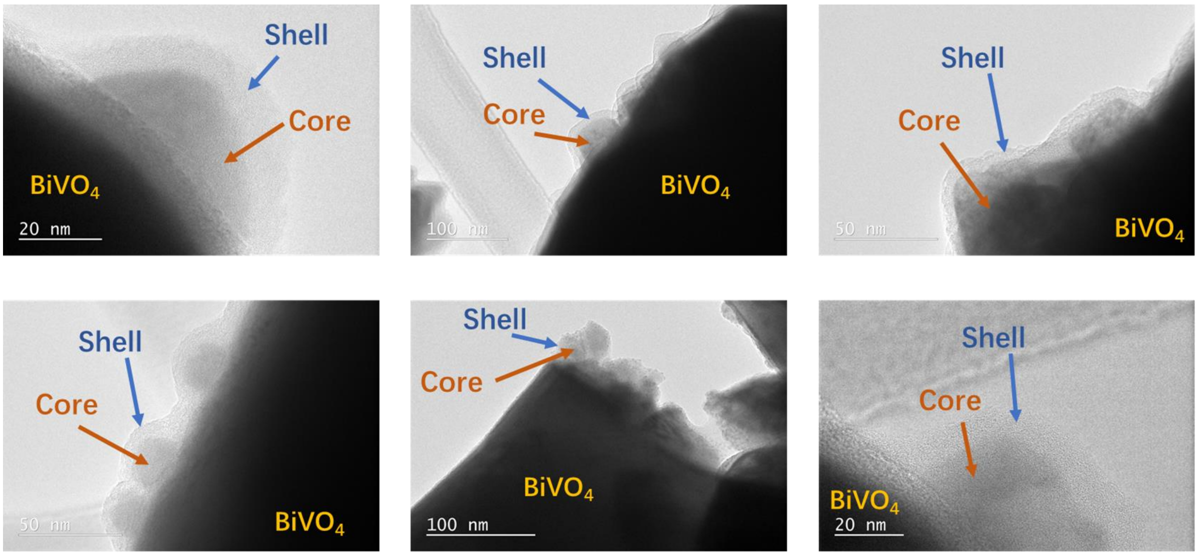

Figure S14 (Continues). (d) HR-TEM micrographs for the $BiVO_4/Ni_{1.5}Co_{0.5}P$ photoanodes prepared in this work after a stability test (Ni_2P: 00-003-0953).

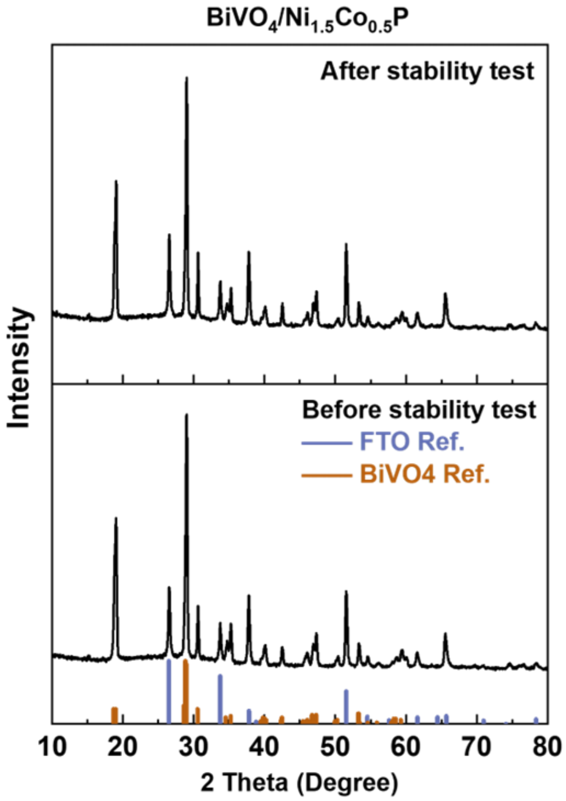

Figure S15. XRD patterns of $BiVO_4/Ni_{1.5}Co_{0.5}P$ before and after running the OER at +0.8 V_{RHE} for 8 h in a 1 M KB buffer electrolyte (pH 9).

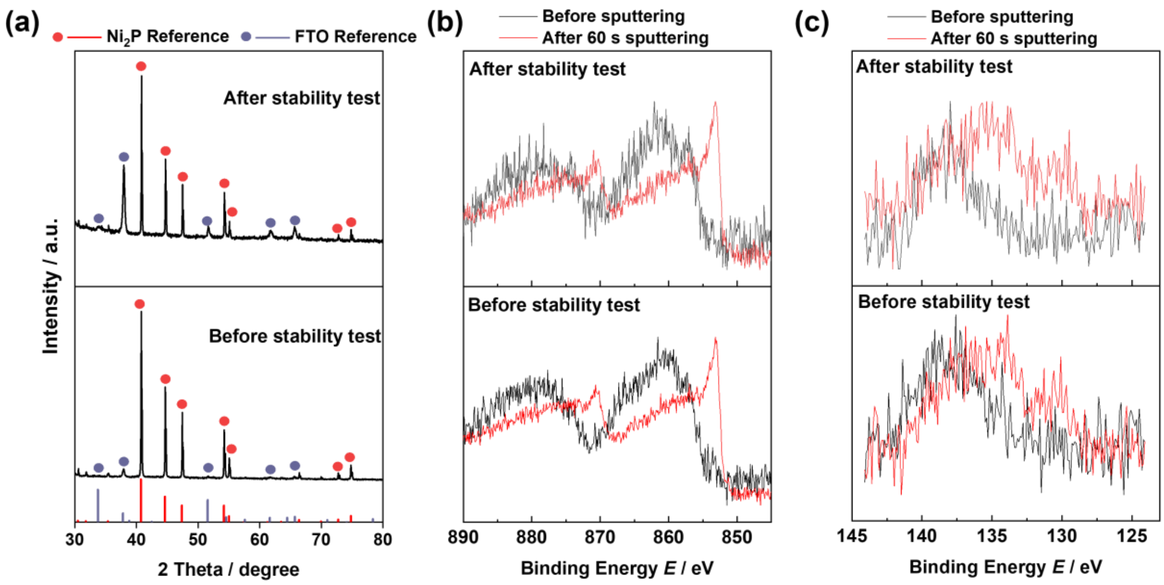

Figure S16. Characterization of $Ni_{1.5}Co_{0.5}P$ drop-cast on FTO-coated glass (for XRD) or carbon paper (for XPS) before and after running the OER at +1.8 V_{RHE} for 2 h in a 1 M KOH electrolyte (pH 13.7). Drop casting was carried out by dispersing the powder in an IPA/water solution containing Nafion binder. (a) XRD patterns and (b)/(c) Ni 2*p* (b) and P 2*p* (c) high-resolution depth-profiling XPS measurements. Note that since F cannot be present in XPS depth-profiling measurements, Ni1.5Co0.5P was drop-casted on carbon paper without the Nafion binder for these measurements. These measurements could not be done with enough resolution directly on $BiVO_4/Ni_{1.5}Co_{0.5}P$ because metal phosphides are only loaded on $BiVO_4$ in minute amounts to avoid parasitic absorption.

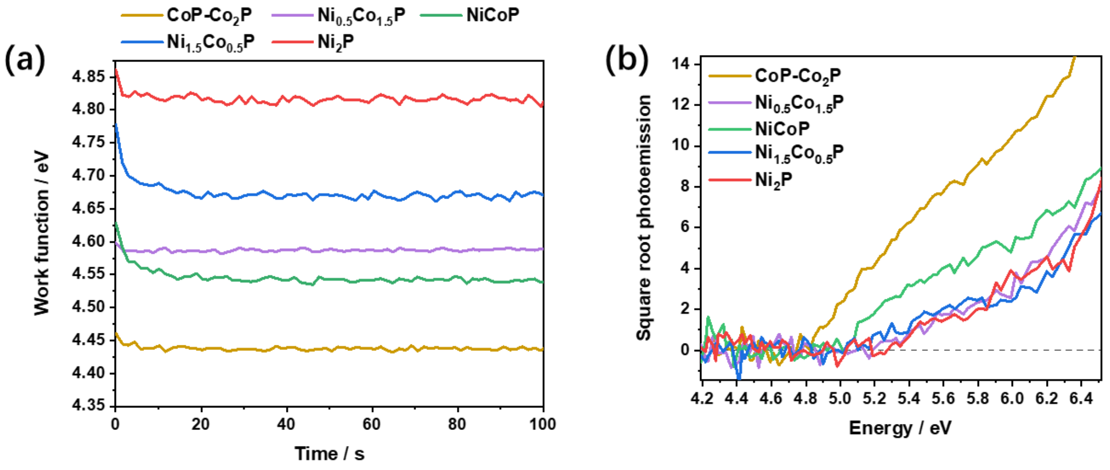

Figure S17. (a) Dark work functions of the metal phosphides prepared in this work measured for 100 s using a Kelvin probe and (b) square root of the photoemission as a function of energy measured using ambient photoemission spectroscopy.

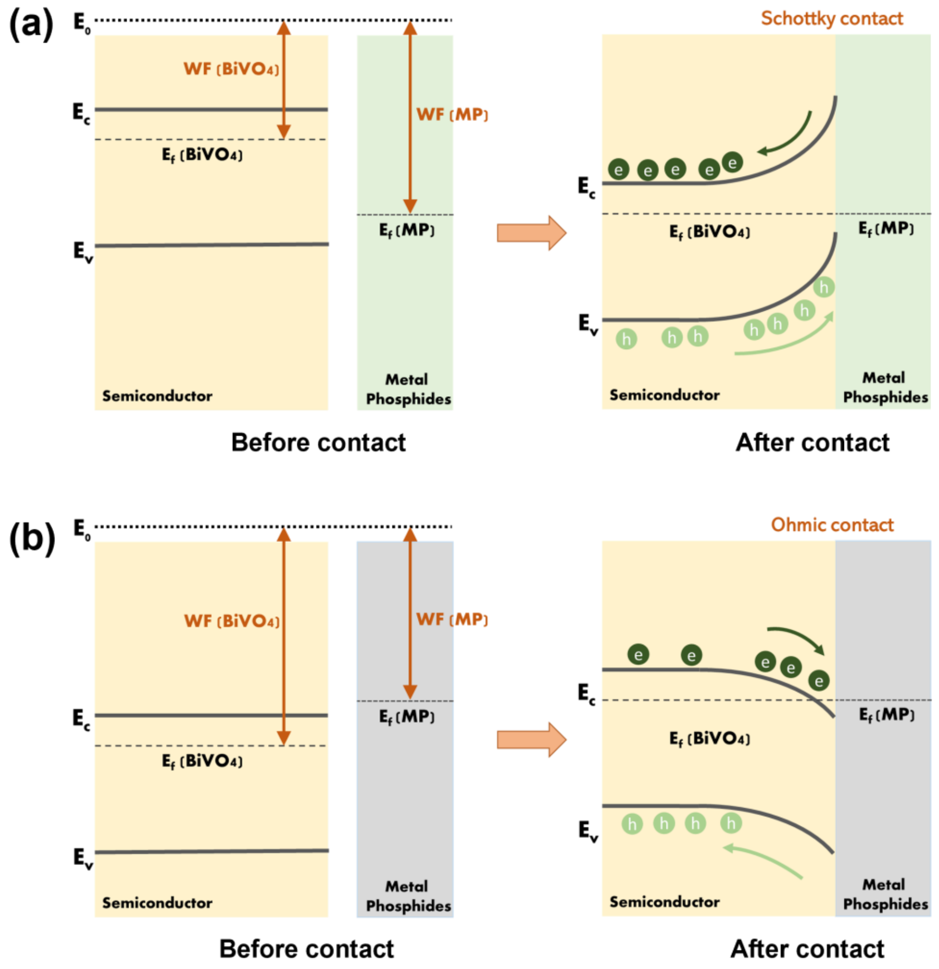

Figure S18. Schematic illustration of the interfacial band bending for Schottky (a) and Ohmic (b) contacts.

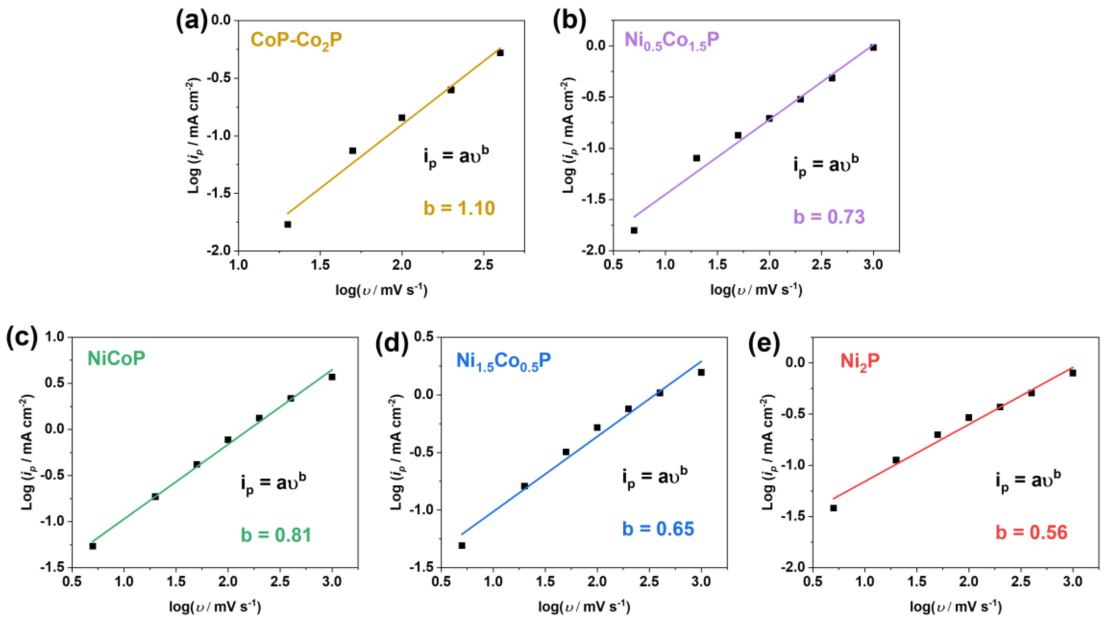

Figure S19. Linear relationship between $\log I_p$ and $\log v$ obtained from the CV measurements on the metal phosphides prepared in this work performed at various scan rates.

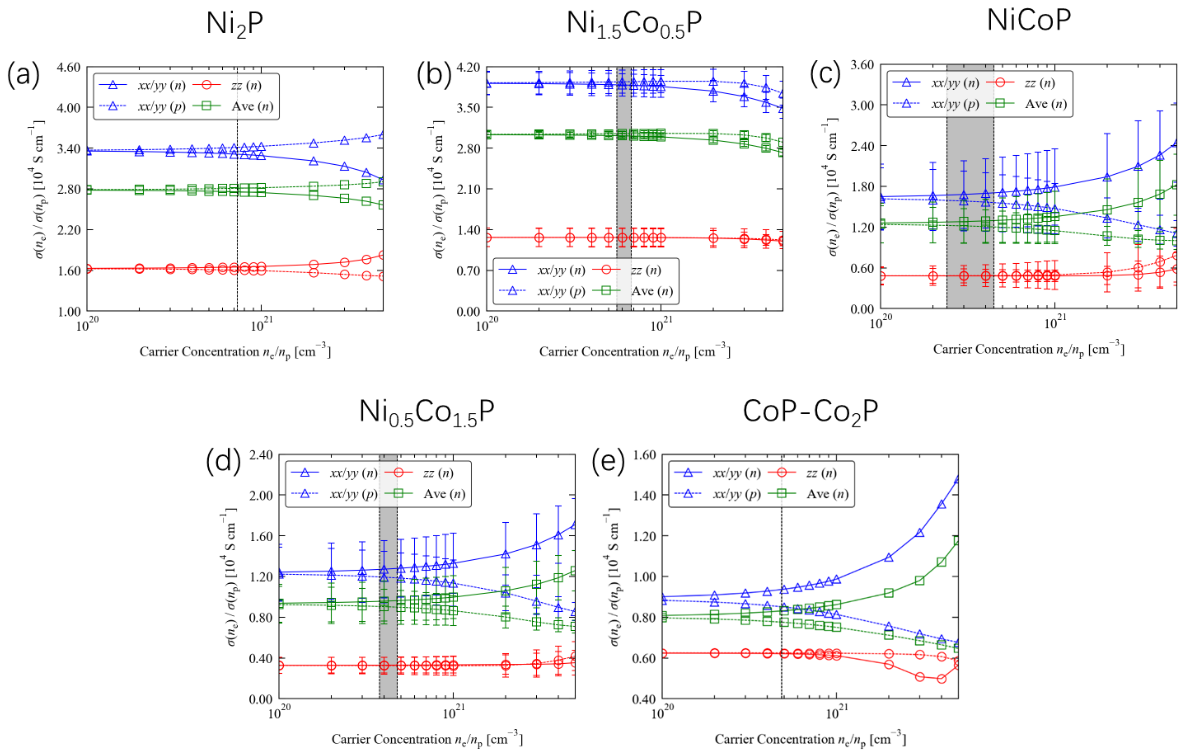

Figure S20. Predicted conductivity σ of the (Ni$_{1\text{-}x}$Co$_x$)P alloys (x = 0, 0.25, 0.5, 0.75 and 1) as a function of carrier concentration obtained from density-functional theory calculations. Values are shown for hole (p) and electron (n) carriers along the x/y directions $(\sigma_{xx} + \sigma_{yy})/2$ and z direction together with the average $\sigma_{\mathrm{ave}} = (\sigma_{xx} + \sigma_{yy} + \sigma_{zz})/3$.

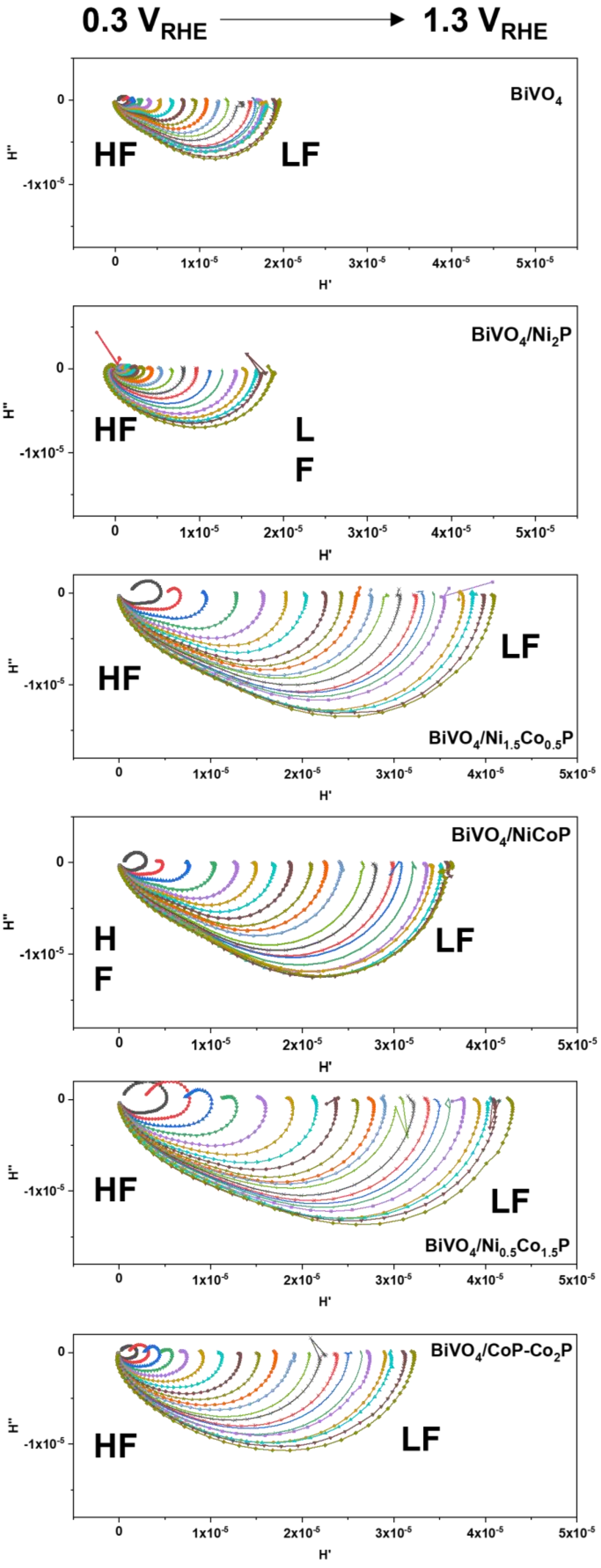

Figure S21. IMPS measurements on the $BiVO_4$ and $BiVO_4$/metal phosphide photoanodes prepared in this work.

(a)

	τ / ms	g / Ω s-1
1	0.5	0.8
2	1	0.5
3	1000	-0.4

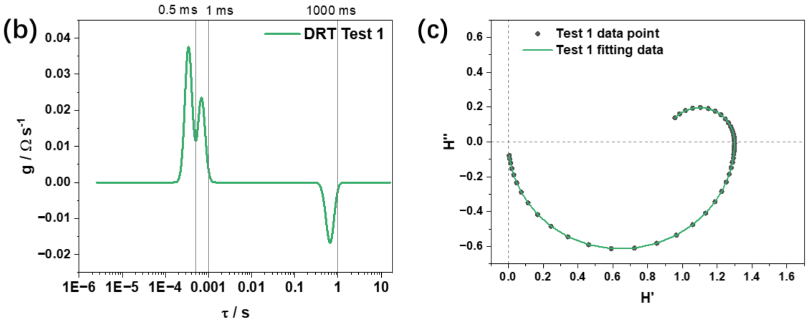

(d)

	τ / ms	g / Ω s-1
1	0.5	0.8
2	1	0.5
3	20	0.3
4	1000	-0.4

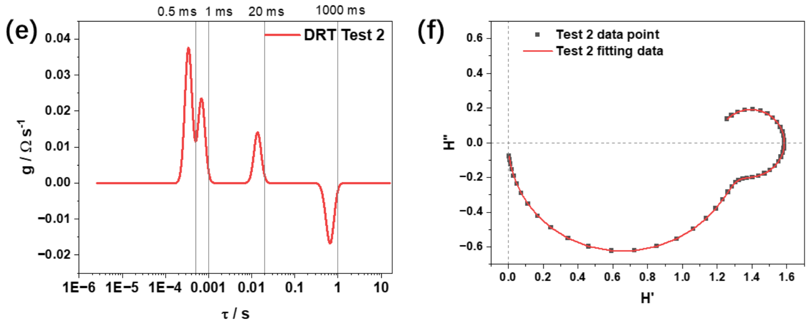

Figure S22. (a)-(f) Pre-set parameters for validation of the distribution of relaxation times (DRT) model, DRT plots, and comparison of simulated and fitted data for (a)-(c) Test 1 and (d)-(f) Test 2.

References:

(1) Kim, T. W.; Choi, K. S. Nanoporous $BiVO_4$ Photoanodes with Dual-Layer Oxygen Evolution Catalysts for Solar Water Splitting. *Science* **2014**, *343* (6174), 990–994. https://doi.org/10.1126/science.1246913.

(2) Kresse, G.; Hafner, J. *Ab Initio* Molecular Dynamics for Liquid Metals. *Phys. Rev. B* **1993**, *47*, 558(R)-561(R).

(3) Jain, A.; Ong, S. P.; Hautier, G.; Chen, W.; Richards, W. D.; Dacek, S.; Cholia, S.; Gunter, D.; Skinner, D.; Ceder, G.; et al. Commentary: The Materials Project: A Materials Genome Approach to Accelerating Materials Innovation. *APL Mater.* **2013**, *1*, 011002. https://doi.org/10.1063/1.4812323.

(4) Perdew, J. P.; Ruzsinszky, A.; Csonka, G. I.; Vydrov, O. A.; Scuseria, G. E.; Constantin, L. A.; Zhou, X.; Burke, K. Restoring the Density-Gradient Expansion for Exchange in Solids and Surfaces. *Phys. Rev. Lett.* **2008**, *100* (13), 136406. https://doi.org/10.1103/PhysRevLett.100.136406.

(5) Dudarev, S. L.; Botton, G. A.; Savrasov, S. Y.; Humph`reys, C. J.; Sutton, A. P. Electron-Energy-Loss Spectra and the Structural Stability of Nickel Oxide: An LSDA+U Study. *Phys. Rev. B* **1998**, *57* (3), 1505–1509. https://doi.org/10.1103/PhysRevB.57.1505.

(6) Wang, M.; Navrotsky, A. Enthalpy of Formation of $LiNiO_2$, $LiCoO_2$ and Their Solid Solution, $LiNi_{1-x}Co_xO_2$. *Solid State Ionics* **2004**, *166* (1–2), 167–173. https://doi.org/10.1016/J.SSI.2003.11.004.

(7) Grimme, S.; Antony, J.; Ehrlich, S.; Krieg, H. A Consistent and Accurate *Ab Initio* Parametrization of Density Functional Dispersion Correction (DFT-D) for the 94 Elements H-Pu. *J. Chem. Phys.* **2010**, *132* (15), 154104. https://doi.org/10.1063/1.3382344.

(8) Blöchl, P. E. Projector Augmented-Wave Method. *Phys. Rev. B* **1994**, *50* (24), 17953–17979. https://doi.org/10.1103/PhysRevB.50.17953.

(9) Kresse, G.; Joubert, D. From Ultrasoft Pseudopotentials to the Projector Augmented-Wave Method. *Phys. Rev. B* **1999**, *59* (3), 1758–1775. https://doi.org/10.1103/PhysRevB.59.1758.

(10) Monkhorst, H. J.; Pack, J. D. Special Points for Brillouin-Zone Integrations. *Phys. Rev. B* **1976**, *13* (12), 5188–5192. https://doi.org/10.1103/PhysRevB.13.5188.

(11) Ganose, A. M.; Park, J.; Faghaninia, A.; Woods-Robinson, R.; Persson, K. A.; Jain, A. Efficient Calculation of Carrier Scattering Rates from First Principles. *Nat. Commun.* **2021**, *12* (1), 1–9. https://doi.org/10.1038/S41467-021-22440-5.

(12) Faghaninia, A.; Yu, G.; Aydemir, U.; Wood, M.; Chen, W.; Rignanese, G.-M.; Snyder, G. J.; Hautier, G.; Jain, A. A Computational Assessment of the Electronic, Thermoelectric, and Defect Properties of Bournonite ($CuPbSbS_3$) and Related Substitutions. *Phys. Chem. Chem. Phys.* **2017**, *19* (9), 6743–6756. https://doi.org/10.1039/C7CP00437K.

(13) Grau-Crespo, R.; Hamad, S.; Catlow, C. R. A.; de Leeuw, N. H. Symmetry-Adapted Configurational Modelling of Fractional Site Occupancy in Solids. *J. Phys. Condens. Matter* **2007**, *19* (25), 256201. https://doi.org/10.1088/0953-8984/19/25/256201.

(14) Gunn, D. S. D.; Skelton, J. M.; Burton, L. A.; Metz, S.; Parker, S. C. Thermodynamics, Electronic Structure, and Vibrational Properties of $Sn_n(S_{1-x}Se_x)_m$ Solid Solutions for Energy Applications. *Chem. Mater.* **2019**, *31* (10), 3672–3685. https://doi.org/10.1021/acs.chemmater.9b00362.

(15) Tung, R. T. Recent Advances in Schottky Barrier Concepts. *Mater. Sci. Eng. R Reports* **2001**, *35* (1–3), 1–138. https://doi.org/10.1016/S0927-796X(01)00037-7.

(16) Jian, J.; Xu, Y.; Yang, X.; Liu, W.; Fu, M.; Yu, H.; Xu, F.; Feng, F.; Jia, L.; Friedrich, D.; et al. Embedding Laser Generated Nanocrystals in $BiVO_4$ Photoanode for Efficient Photoelectrochemical Water Splitting. *Nat. Commun. 2019 101* **2019**, *10* (1), 1–9. https://doi.org/10.1038/s41467-019-10543-z.

(17) Ma, Y.; Pendlebury, S. R.; Reynal, A.; Formal, F. Le; Durrant, J. R. Dynamics of Photogenerated Holes in Undoped $BiVO_4$ Photoanodes for Solar Water Oxidation. *Chem. Sci.* **2014**, *5* (8), 2964–2973. https://doi.org/10.1039/C4SC00469H.

(18) Sze, S. M.; Ng, K. K. *Physics of Semiconductor Devices*; John Wiley & Sons, Inc.: Hoboken, NJ, USA, 2006. https://doi.org/10.1002/0470068329.
